# Multilevel Factors and Indicators of Atypical Neurodevelopment During Early Infancy in Japan: Prospective, Longitudinal, Observational Study

**DOI:** 10.2196/58337

**Published:** 2025-04-04

**Authors:** Daigo Kato, Akiko Okuno, Tetsuo Ishikawa, Shoji Itakura, Shinji Oguchi, Yoshiyuki Kasahara, Kenji Kanenishi, Yuzo Kitadai, Yoshitaka Kimura, Naoki Shimojo, Kazushige Nakahara, Akiko Hanai, Hiromichi Hamada, Haruta Mogami, Seiichi Morokuma, Kazuhiro Sakurada, Yukuo Konishi, Eiryo Kawakami

**Affiliations:** 1 Department of Artificial Intelligence Medicine, Graduate School of Medicine, Chiba University, Chiba, Japan; 2 Department of Pediatrics, Graduate School of Medicine, Chiba University, Chiba, Japan; 3 Advanced Data Science Project, RIKEN Information R&D and Strategy Headquarters, 1-7-22 Suehiro-cho, Tsurumi-ku, Yokohama, 230-0045, Japan, 81 45 503 7012, 81 45 503 7010; 4 Center for Baby Science, Doshisha University, Kyoto, Japan; 5 Department of Extended Intelligence for Medicine, The Ishii-Ishibashi Laboratory, Keio University School of Medicine, Shinjuku, Japan; 6 Collective Intelligence Research Laboratory, Graduate School of Arts and Sciences, The University of Tokyo, Meguro, Japan; 7 Department of Maternal and Fetal Therapeutics, Graduate School of Medicine, Tohoku University, Sendai, Japan; 8 Department of Perinatology and Gynecology, Graduate School of Medicine, Kagawa University, Kita, Japan; 9 Department of Obstetrics, Fukuoka Children's Hospital, Fukuoka, Japan; 10 Center for Preventive Medical Sciences, Chiba University, Chiba, Japan; 11 Department of Obstetrics and Gynecology, Graduate School of Medical Sciences, Kyushu University, Fukuoka, Japan; 12 Department of Gynecology and Obstetrics, Kyoto University Graduate School of Medicine, Kyoto, Japan; 13 Department of Health Sciences, Graduate School of Medical Sciences, Kyushu University, Fukuoka, Japan

**Keywords:** early developmental signs, neurodevelopmental screening, risk factors, prediction, early intervention, longitudinal study

## Abstract

**Background:**

The early identification of developmental concerns requires understanding individual differences that may represent early signs of neurodevelopmental conditions. However, few studies have longitudinally examined how child and maternal factors interact to shape these early developmental characteristics.

**Objective:**

We aim to identify factors from the perinatal to infant periods associated with early developmental characteristics that may precede formal diagnoses and propose a method for evaluating individual differences in neurodevelopmental trajectories.

**Methods:**

A prospective longitudinal observational study of 147 mother-child pairs was conducted from gestation to 12 months post partum. Assessments included prenatal questionnaires and blood collection, cord blood at delivery, and postpartum questionnaires at 1, 6, and 12 months. The Modified Checklist for Autism in Toddlers (M-CHAT) was used to evaluate developmental characteristics that might indicate early signs of atypical neurodevelopment. Polychoric or polyserial correlation coefficients assessed relationships between M-CHAT scores and longitudinal variables. L2-regularized logistic regression and Shapley Additive Explanations predicted M-CHAT scores and determined feature contributions.

**Results:**

Twenty-one factors (4 prenatal, 3 at birth, and 14 postnatal) showed significant associations with M-CHAT scores (adjusted *P* values<.05). The predictive accuracy for M-CHAT scores demonstrated reasonable predictive accuracy (area under the receiver operating characteristic curve=0.79). Key predictors included infant sleep status after 6 months (nighttime sleep duration, bedtime, and difficulties falling asleep), maternal Kessler Psychological Distress Scale scores, and Mother-to-Infant Bonding Scale scores after late gestation.

**Conclusion:**

Maternal psychological distress, mother-infant bonding, and infant sleep patterns were identified as significant predictors of early developmental characteristics that may indicate emerging developmental concerns. This study advances our understanding of early developmental assessment by providing a novel approach to identifying and evaluating early indicators of atypical neurodevelopment.

## Introduction

Early identification of neurodevelopmental disorders and timely intervention are crucial for optimizing developmental outcomes in children [1]. Children exhibit considerable individual differences in their developmental trajectories during the first year of life, particularly in preverbal social behavior skills [2]. These differences have emerged as crucial areas of study in neurodevelopmental research, as some behavioral characteristics observed during infancy may represent early indicators of developmental concerns [3-5]. Similar to how the concept of mild cognitive impairment has facilitated early intervention in preclinical stages of dementia [6], identifying early indicators of developmental concerns may enable more timely and effective support strategies. However, the complex nature of early development, with its inherent variability and multiple influencing factors, has made it challenging to identify and interpret these early indicators reliably.

Multiple factors influence early childhood neurodevelopment, including prenatal conditions such as maternal immune activation [7], stress [8], and nutritional status, as well as postnatal factors including parental depression [9,10] and early life experiences [11]. Recent research has also highlighted the importance of biological factors such as cytokine profiles [12-14] and sleep-wake rhythms [15-18] in early neurodevelopment. While these factors have been studied individually, their complex interactions across time and their cumulative impact on neurodevelopmental trajectories remain poorly understood.

This study is an exploratory longitudinal investigation conducted in Japan, following mothers and children from the perinatal period (20 weeks of gestation) to the end of infancy (12 months of age). Using an innovative explainable artificial intelligence methodology, we aimed to visualize and analyze how various longitudinal factors interact to influence individual neurodevelopmental trajectories during infancy. Specifically, we assessed preverbal social behavior skills at 12 months of age as potential early indicators of developmental concerns using the Modified Checklist for Autism in Toddlers (M-CHAT) [3,5], and systematically evaluated how multiple observed factors throughout the first year of life contributed to these developmental characteristics. This approach enables a comprehensive understanding of the temporal dynamics and relative importance of various factors in shaping early neurodevelopmental trajectories, potentially identifying key time points and factors for early intervention.

## Methods

### Recruitment

This study is part of an ongoing multicenter Japanese longitudinal research conducted by 6 collaborating institutions—namely, Kyushu University Graduate School of Medicine, Kagawa University School of Medicine, Tohoku University Graduate School of Medicine, Kyoto University School of Medicine, Fukuoka City Children’s Hospital, and RIKEN. Pregnant women were recruited between October 2018 and June 2020 at 5 of the collaborating institutions (excluding RIKEN). Overall, 200 women who fulfilled the inclusion criteria (aged ≥20 years, pregnant with a gestational period of less than 26 completed weeks, and fetus in stable condition) agreed to participate. The exclusion criteria were the requirement for proxy consent or the inability to provide voluntary consent. These criteria were established by the Ethical Guidelines for Medical and Health Research Involving Human Subjects to ensure that severe comorbidities or extreme factors would not influence the results of this study. Participants were followed up by visiting the affiliated facilities at midgestation (24‐26 weeks), late gestation (34‐38 weeks), at birth, and 1 month post partum. Their children were enrolled at birth and followed up by mailing questionnaires at 1, 6, and 12 months of age. By November 2022, a total of 147 participants and their children had completed the 12-month follow-up. Two patients were excluded due to meeting the exclusion criteria for chromosomal abnormalities: 1 with trisomy 21 and another with an unspecified chromosomal abnormality. The third excluded patient was born at 25 weeks’ gestation weighing 734 g, thus meeting both the criteria for very low birth weight (<1000 g) and extreme preterm birth (<30 weeks). These 3 cases were consequently removed from the study population. This study was prospectively registered in the UMIN Clinical Trials Registry (UMIN000034837) on November 9, 2018. While registered in a clinical trial registry, this research is an exploratory study, not a clinical trial. It uses nonpredefined analytical methods, which distinguishes it from traditional clinical trials with preset end points and analysis plans. The registration was carried out for transparency, despite the exploratory nature precluding a detailed prespecified analysis plan. This study was reported according to the STROBE (Strengthening the Reporting of Observational Studies in Epidemiology) guidelines.

### Maternal Assessment

Peripheral blood collected at mid- and late gestation and cord blood at birth was used to measure levels of 1,25-dihydroxyvitamin D, 25-hydroxyvitamin D, melatonin, and cytokines (interleukin [IL]-17A, IL-10, IL-1β, IL-6, and tumor necrosis factor). The participating mothers completed a series of questionnaires at mid- and late gestation and at 1 month post delivery (Multimedia Appendix 1). Maternal hematological biomarkers (vitamin D, melatonin, and cytokines) were measured from blood samples collected during prenatal check-ups and from umbilical cord blood at delivery. While prenatal check-ups typically occur during daytime hours, we did not standardize the exact timing of blood collection. Similarly, as birth times vary unpredictably, umbilical cord blood collection times were inconsistent across subjects. Maternal sleep status was evaluated using the Pittsburgh Sleep Quality Index [19] to assess the quality of sleep and the 3D Sleep Scale (3DSS) [20] to assess sleep regularity (the “sleep phase” item). The Edinburgh Postnatal Depression Scale (EPDS) [21] and Kessler Psychological Distress Scale (K6) [22] were used to assess depression and anxiety disorders, respectively, during pregnancy and post partum. The late gestation questionnaire also included the Autism-Spectrum Quotient Japanese short version (AQ-J-10) [23]. At 1 month post partum, the Japanese version of the Mother-to-Infant Bonding Scale (MIBS-J) [24] was used to assess the mothers’ feelings toward their babies.

### Infant Assessment and Outcomes

When the children were 1, 6, and 12 months of age, mothers completed questionnaires regarding the children’s sleep status (Multimedia Appendix 2). To assess early developmental characteristics in infants, we focused on the acquisition of standard preverbal social behavioral skills and the presence of behaviors commonly associated with autism spectrum disorder (ASD). Specifically, we administered the Japanese version of the M-CHAT at 12 months of age to systematically evaluate these early developmental characteristics. The M-CHAT—a parent-completed dichotomous questionnaire designed for children aged 16‐30 months—is an effective primary screening tool for ASD and other developmental concerns in the general population [3,5]. In our study, we aimed to identify children showing potential developmental concerns at an earlier stage, specifically at 12 months of age. To achieve this, we developed a unique 10-item version of the M-CHAT, informed by age-specific achievement rates observed in a separate observational study of the general Japanese population. Given that prelinguistic social behaviors vary significantly with age, we included in our analysis 4 age-independent ASD-specific behavioral abnormalities (Q11, Q18, Q20, and Q22) and 6 prelinguistic social behaviors (Q1, Q2, Q4, Q10, Q12, and Q14) that are reported to be consistently achieved by 12 months of age in the general population [4]. The items included were as follows: (Q1) “enjoying being swung,” (Q2) “interest in other children,” (Q4) “enjoying peek-a-boo,” (Q10) “eye contact,” (Q11) “oversensitive to noise,” (Q12) “response to smile,” (Q14) “response to name,” (Q18) “unusual finger movement,” (Q20) “wonder if deaf,” and (Q22) “stares at nothing.” Children who fulfilled any of the 10 total M-CHAT items (M-CHAT score ≥1 point) were defined as the M-CHAT positive group.

### Statistical Analysis

Descriptive statistics were used to investigate the characteristics of the participating mothers and their infants. Continuous variables—listed as the median and IQR—included maternal age, 1,25-dihydroxyvitamin D, 25-hydroxyvitamin D, melatonin, IL-17A, IL-10, IL-1β, IL-6, tumor necrosis factor, 3DSS (phase), Pittsburgh Sleep Quality Index global score (PSQIG), EPDS, K6, AQ-J-10, MIBS-J, and umbilical artery blood pH. Categorical variables—listed as frequencies and percentages—included the child’s sex, maternal smoking history, educational level of parents, annual household income, gestational age at birth, birth weight, Apgar score at 5 minutes, delivery type, answers to sleep-related questions at each period, and the M-CHAT score. Thereafter, the correlation coefficients between the variables were calculated considering the aforementioned categorical variables and 3DSS (phase), PSQIG, EPDS, K6, AQ-J-10, and MIBS-J as ordinal variables. Polyserial correlation coefficients were computed for the relationships between ordinal and continuous variables; further, polychoric correlation coefficients were computed for the relationships between ordinal variables. These polychoric and polyserial correlation coefficients, as well as Pearson correlation coefficients for continuous variable pairs, were calculated using the lavCor function from the *lavaan* package in R software (R Foundation). Wald tests were performed to assess the statistical significance of each correlation. To address the issue of multiple comparisons arising from these correlation tests, false discovery rate correction [25] was subsequently applied to the resulting *P* values. This was performed using the p.adjust() function in R software. Adjusted *P* values (p.adjust) of <.05 were considered statistically significant.

### Correlation Analysis

In this study, the relationships between variables affecting the M-CHAT score were represented by network structure. The nodes were variables that showed significant polychoric or polyserial correlations with the M-CHAT score (|r|≥.2, p.adjust <.05). The nodes were arranged according to the data collection period.

### Classification Model for the M-CHAT Positive Group

We implemented a classification model using a logistic regression algorithm with potential regularization in the scikit-learn Python package (version 3.1.2) to predict the M-CHAT positive group in infants aged 12 months. We used features of maternal and child demographics, laboratory values, and questionnaire results from the midgestation to 12 months post partum that exhibited a significant correlation (p.adjust<.05) and |r|≥.2 with the M-CHAT score. This model, overall, used 20 features. The data obtained at 12 months of age had missing values, which were completed with missForest—a modern method based on random forests that efficiently addresses missing data imputation among multivariate data without cross-validating with the test data [26]. This method involves inherent randomness, potentially leading to variability in imputed datasets. In our study, we did not set a fixed seed during the missForest imputation. The mother-child dataset was divided into a training set (N=100) for training the prediction model and a test set (N=44) for evaluating the model’s performance by using the train_test_split function in scikit-learn. To address the imbalanced nature of our dataset, we used the “stratify” parameter in the train_test_split function, specifying the binary M-CHAT outcome (positive or negative) as the stratification variable. This approach ensured that the distribution of M-CHAT outcomes was maintained across both the training and test sets. The models were optimized by adapting a grid search method for the hyperparameter “C (regularization strength; 10^−5^, 10^−4^, 10^−3^, 10^−2^, 10^−1^, 1, 10, 10^2^, 10^3^, 10^4^, or 10^5^),” “penalty (norm of regularization; L1, L2, elastic net, or none),” and “solver (algorithm in optimization; “newton-cg,” “lbfgs,” “liblinear,” “sag,” “saga”).” When specifying elastic net as the penalty, we validated by varying the l1_ratio from 0 to 1 in steps of 0.1. The performance of the logistic regression model was evaluated on the test set after ensuring missing data imputation. The model outputs class probabilities to predict the M-CHAT positive (M-CHAT score≥1) group. The optimal threshold for the model was determined by the Youden index.

The model’s performance was evaluated using the area under the receiver operating characteristic and precision-recall curves. The relative importance of each feature for predicting M-CHAT scores was examined and visualized using Shapley Additive Explanations (SHAP) [27]. SHAP is a model-agnostic, game-theoretic, unified method that computes a Shapley value to account for each feature’s contribution to a particular forecast. SHAP values are computed using an additive feature attribution method to approximate the Shapley value of the model’s conditional expectation function. LinearExplainer [28] was used to calculate SHAP values for the logistic regression model. For the decision plotting that simulates the M-CHAT prediction pathway of each sample using SHAP, we specifically used samples with higher M-CHAT scores (M-CHAT scores 2‐4) within the M-CHAT positive group to focus on cases that might represent early developmental concerns.

Data analysis was performed using the open-source software R (version 4.1.2) and Python (version 3.8.5; Python Software Foundation). The correlation analysis results were visualized using Cytoscape (version 3.9.1; Cytoscape Consortium).

### Ethical Considerations

This study was approved by the Ethics Review Committee on Research Involving Human Subjects at Doshisha University (No. 22063) and, subsequently, by the Ethics Review Committees at each affiliated institution (No. 22064). Written informed consent was provided by each pregnant woman at the time of the visit at the affiliated facility. Data was deidentified immediately upon collection. A secure, separate database is maintained to connect study IDs with participant details, accessible only to authorized personnel. The research description clearly states the following points: (1) women are free to participate in the research and withdraw their consent, (2) refusal of participation in the research has no implications per disadvantages in medical care, and (3) participation in the research entails no increased cost burden or rewards.

## Results

### Description of the Sample

The characteristics and results for the 144 mother-child pairs included in the final analysis are presented in Table 1 and Multimedia Appendix 2. These maternal background and perinatal data were obtained by combining information from medical records and parents’ reports up to the first month postpartum check-up. The median maternal age was 36 (IQR 32-29) years, and 3 (N=144, 2%) mothers had a smoking history during their current pregnancy. The most prevalent educational level for both parents was a university degree (48/144, 33% mothers and 64/144, 44% fathers). Annual household incomes varied among the participants, with the most common bracket being 4 to 6 million yen (approximately US $36,000 to US $56,000, based on a currency exchange rate of US $1=JP ¥107-112 that was applicable during the study period) per year, reported by 46 (N=142, 32% of the population) families. Of the children, 86 (N=144, 60%) were female, 129 (N=144, 90%) were born at 37 weeks or later, and 3 (N=144, 2%) were born between 30 and 33 weeks. Birth weight ranged from 3000 g to 3999 g in 122 (N=144, 85%) children; all 5-minute Apgar scores were 7 (N=144) or higher. The median umbilical artery blood gas pH was 7.29 (IQR 7.26‐7.33). The delivery type was vaginal in 87 (N=144, 60%) cases (Table 1). Throughout the pregnancy, the 3DSS (phase) median score was 10 (IQR 9-12). From midgestation to 1-month postpartum, PSQIG exhibited an increasing trend with median scores of 5 (IQR 3-7), 6 (IQR 4.5-9), and 8 (IQR 6-10), respectively. This indicates that mothers’ sleep conditions tended to worsen during the period up to 1 month after childbirth. The median EPDS scores were relatively low across all 3 time points: 3 points in midpregnancy, 4 points in late pregnancy, and 2 points at 1 month post partum. Similarly, K6 scores remained low at 1, 2, and 1, respectively. The median AQ-J-10 score at late gestation was 2 (IQR 1-3), and the median MIBS-J score at 1 month post partum was 1 (IQR 0-3; Multimedia Appendix 2).

**Table 1.** Demographic data for the 144 mother-child pairs included in the final analysis.

Characteristics		Longitudinal group
Maternal age (years; N=144), median (IQR)		36 (32-39)
Child’s sex (N=144), n (%)		
	Female	86 (60)
	Male	58 (40)
Maternal smoking status (N=144), n (%)		
	Never or exsmoker	141 (98)
	Current smoker	3 (2.1)
Maternal education (N=144), n (%)		
	Junior high	3 (2.1)
	High school	28 (19)
	Technical college	5 (3.5)
	College	33 (23)
	Junior college	17 (12)
	University	48 (33)
	Master’s degree	10 (6.9)
Paternal education (N=144), n (%)		
	Junior high	7 (4.9)
	High school	37 (26)
	Technical college	3 (2.1)
	College	15 (10)
	Junior college	0 (0)
	University	64 (44)
	Master’s degree	18 (12)
Annual household income (JP ¥^a^; n=142), n (%)		
	<2 million	1 (0.7)
	2‐3.9 million	29 (20)
	4‐5.9 million	46 (32)
	6‐7.9 million	32 (23)
	8‐9.9 million	19 (13)
	10‐11.9 million	11 (7.7)
	12‐14.9 million	1 (0.7)
	15‐19.9 million	3 (2.1)
Gestational age at birth (wk; N=144), n (%)		
	30‐32	3 (2.1)
	33‐36	12 (8.3)
	≥37	129 (90)
Birth weight (g; N=144), n (%)		
	<1500	1 (0.7)
	1500‐2999	20 (14)
	3000‐3999	122 (85)
	≥4000	1 (0.7)
Apgar score at 5 min (N=144), n (%)		
	7	1 (0.7)
	8	16 (11)
	9	116 (81)
	10	11 (7.6)
Umbilical artery blood pH (n=140), median (IQR)		7.29 (7.26‐7.33)
Type of delivery (N=144), n (%)		
	Vaginal	87 (60)
	Cesarean	57 (40)

a A currency exchange rate of 107-112 JP ¥=US $1 was applicable during the study period.

### The Distribution of the M-CHAT Score Among Children Aged 12 Months

The distribution of the M-CHAT scores among the 144 children aged 12 months was as follows: 105 (73%) children scored 0, 30 (21%) scored 1, 5 (3.5%) scored 2, 3 (2.1%) scored 3, and 1 (1%) scored 4. All 6 preverbal social behavior items (Q1, Q2, Q4, Q10, Q12, and Q14) used to score the M-CHAT in this study exhibited achievement rates of 95% or higher. Specifically, for the question on smiling response (Q12), only 1 parent reported that their child did not smile back when smiled at. Similarly, regarding concerns of hearing impairment (Q20), only 1 parent expressed concern that their child might be enduring hearing difficulties. Of the 39 children who scored 1 or more, 29 (74%) received scores for age-independent and ASD-specific behaviors (particularly Q11, Q18, and Q22; Multimedia Appendix 3).

### Interrelationships Between M-CHAT Score and Each Variable

**Figure 1.**
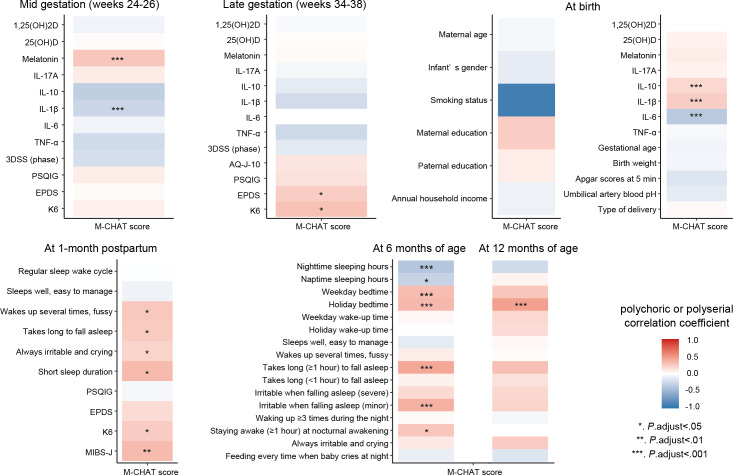
Heatmap of correlations between M-CHAT and each other variable. 1,25(OH)2D: 1,25-dihydroxyvitamin D; 25(OH)D: 25-hydroxyvitamin D; 3DSS: 3D Sleep Scale; AQ-J-10: Autism-Spectrum Quotient Japanese short version; EPDS: Edinburgh Postnatal Depression Scale; IL: interleukin; K6: Kessler Psychological Distress Scale; M-CHAT: Modified Checklist for Autism in Toddlers; MIBS-J: Japanese version of the Mother-to-Infant Bonding Scale; PSQIG: Pittsburgh Sleep Quality Index global score; TNF: tumor necrosis factor.

To examine the relationship between the M-CHAT score and other variables during each period, polychoric or polyserial correlation coefficients were calculated and presented in a heatmap (Figure 1). Using the correlation heatmap, variables that significantly correlated with the M-CHAT score were identified. The M-CHAT score was positively correlated with maternal serum melatonin levels in midgestation and cord blood IL-1β; the M-CHAT score showed a weak positive correlation with cord blood IL-10 (*r*=.18). By contrast, the M-CHAT score exhibited a negative correlation with maternal serum IL-1β levels in midgestation and cord blood IL-6 levels. The M-CHAT score was positively correlated with EPDS and K6 scores in late gestation and with K6 and MIBS-J scores at 1 month post partum. For infant sleep questions, the M-CHAT score was significantly correlated with shorter sleep duration, bedtime, and difficulty in falling asleep (moodiness and required time). Detailed correlation coefficients are presented in Multimedia Appendix 4.

Correlation analysis was performed to determine the interrelationships among factors potentially influencing early developmental characteristics as assessed by M-CHAT scores (Multimedia Appendix 5). Strong positive correlations (*r*≈1.0) were found between cord blood IL-6 and IL-1β levels and between weekday and holiday bedtimes of children aged 6 months. Nodes at late gestation, cord blood, and 1 month post partum were strongly and positively correlated with each other within the same observation period. In children aged 6 months, a negative correlation was found between “nighttime sleeping hours” and “bedtime,” and a positive correlation was found between “staying awake for ≥1 hour at nocturnal awakening” and “takes long (≥1 hour) to fall asleep.” A negative correlation was found between IL-1β levels in the midgestation period and “short sleep duration” in children aged 1 month; a positive correlation was observed between melatonin levels in midgestation and “takes long (≥1 hour) to fall asleep” in children aged 6 months. K6 and EPDS scores in the late gestation were positively correlated with K6 scores in 1 month post partum and also positively correlated with “staying awake for ≥1 hour at nocturnal awakening” in children aged 6 months. “Wakes up several times at night, does not sleep and takes several hands” in children aged 1 month was positively correlated with “takes long (≥1 hour) to fall asleep” in children aged 6 months. “Bedtime” among children aged 6 months was positively correlated with “bedtime on holidays” among children aged 12 months (Multimedia Appendix 5).

### Assessment and Visualization of Factors Contributing to Early Developmental Characteristics

**Figure 2.**
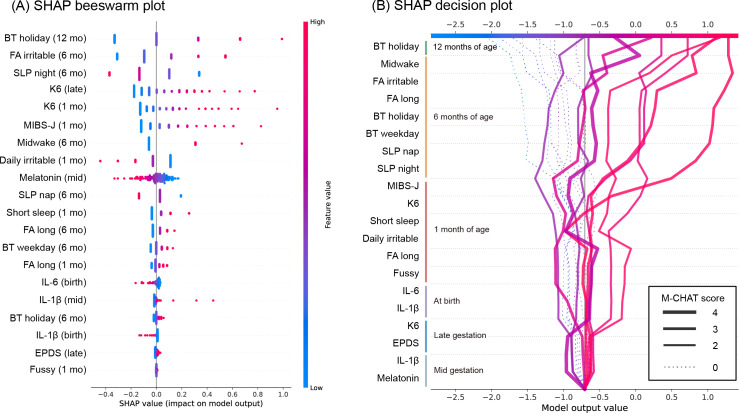
(A) SHapley Additive exPlanations (SHAP) beeswarm plot of the logistic regression model. (B) SHAP decision plot of 9 samples with an M-CHAT score of 0 (random sampling) and 9 samples with an M-CHAT score of 2–4, for a total of 18 samples. BT: bedtime; EPDS: Edinburgh Postnatal Depression Scale; FA: fall asleep; IL: interleukin; K6: Kessler Psychological Distress Scale; M-CHAT: Modified Checklist for Autism in Toddlers; MIBS-J: Japanese version of the Mother-to-Infant Bonding Scale; SHAP: Shapley Additive Explanations; SLP: sleeping hours.

To assess longitudinal influences associated with early developmental characteristics as measured by M-CHAT scores, we conducted feature importance analysis using SHAP on a logistic regression model with L2 regularization (“C”=0.1, “penalty”=”L2,” “solver” = “liblinear,” area under the receiver operating characteristic and precision-recall curves and the confusion matrix shown in Multimedia Appendix 6). The optimal threshold was determined by the Youden index to be 0.32. The logistic regression model attained reasonable predictive performance (area under the receiver operating characteristic curve=0.79, 95% CI 0.62‐0.95; Multimedia Appendix 6). While the point estimate falls within the acceptable range (0.7‐0.8), the CI extends below 0.7, indicating some uncertainty in its discriminative ability. The model’s utility should therefore be interpreted within the context of the dataset and application. The SHAP beeswarm plot (Figure 2A) assesses the contribution of each feature of the total sample to the model. The order of the feature values obtained by SHAP and the contribution of each feature is high, and this ranking is relative, for example, it does not indicate which rank is particularly important. When examining variables in order of their contribution strength, we found high contributions from bedtime at 12 months, nighttime sleep duration and irritability at bedtime at 6 months, and maternal K6 and MIBS-J scores during late pregnancy and 1 month post partum. Specifically, later bedtimes, shorter nighttime sleep duration, higher K6 and MIBS-J scores, and irritability at bedtime promoted classification into the M-CHAT positive group. The characteristics that moderately contributed to such a classification were variables related to the sleep status of children aged 1-6 months and maternal melatonin levels. The 6 characteristics with the lowest influence were predominantly cytokine levels, EPDS score, “wakes up several times at night, does not sleep, and takes several hands” in children aged 1 month, and bedtime for children aged 6 months. The SHAP decision plot (Figure 2B) simulates the decision pathway for predicting classification into the M-CHAT positive or negative (M-CHAT score=0) groups in the order of observation period. All 9 randomly sampled cases with M-CHAT scores of 0 were correctly predicted to be the M-CHAT negative group. In some M-CHAT positive cases, the predictive risk began to increase owing to the deterioration of the maternal K6 and MIBS-J scores and infant sleep status for children aged 6 months. Conversely, there were cases in which the predictive risk did not change for maternal factors but increased rapidly for factors related to the child’s sleep after 6 months of age.

## Discussion

### Temporal Dynamics of Early Developmental Characteristics: Individual Patterns and Contributing Factors

In recent years, the number of cases of neurodevelopmental disorders has increased rapidly, becoming a significant social concern. While genetic factors have been extensively studied, identifying genes that correlate with symptoms alone is insufficient to understand developmental mechanisms. The clarification of how environmental factors interact with genetic predisposition in the developmental process remains an urgent research priority [29].

In this study, we assessed early developmental characteristics through social behavior patterns and analyzed how these patterns emerge during infancy. The SHAP analysis provided a novel approach to assess and visualize how multiple longitudinal factors contribute to individual neurodevelopmental trajectories. Specifically, our analysis focused on cases showing elevated M-CHAT scores (2‐4 points), including those exceeding the conventional ASD screening cutoff value (M-CHAT score of 3+). This analysis revealed distinct temporal patterns in the emergence of early developmental characteristics. Some cases showed an increased risk of M-CHAT positive classification during late gestation, primarily associated with elevated maternal K6 scores. Other cases demonstrated increased risk due to disrupted infant sleep patterns after 6 months of age. Additionally, we observed cases where substantial increases in K6 and Mother-to-Infant Bonding Scale (MIBS) scores at 1-month postpartum markedly elevated the predicted probability of M-CHAT positive classification.

These results indicate that early developmental characteristics associated with elevated M-CHAT scores emerge through several distinct dynamic patterns, involving multiple interacting factors. The SHAP methodology enabled the quantification of each factor’s relative contribution to these patterns. Based on our findings, we suggest that targeted follow-up assessments focusing on maternal anxiety disorders in late gestation and infant sleep characteristics at 6 months—particularly sleep duration, bedtime, irritability upon falling asleep, and extended periods of wakefulness—may be particularly valuable for the early identification of developmental concerns.

### Temporal Relationships Between Early Developmental Characteristics and Longitudinal Factors

Correlation analysis revealed that relatively strong correlations between factors within the same period were observed between similar factors such as maternal physical and mental status, sleep difficulties in children, and inflammatory cytokines. However, correlations between factors with different observation periods were more limited than correlations within the same period. Our analytical method does not pursue a causal relationship.

### Early Sleep Difficulties as Indicators of Emerging Developmental Characteristics

Recent research has established clear interactions between sleep patterns and neurodevelopment in young children. During early brain development, sleep patterns and neural maturation progress in parallel, significantly influencing cognitive performance [16,17]. Notably, studies have demonstrated associations between infant sleep patterns and brain structure, including hippocampal volume [18]. Children who later receive neurodevelopmental disorder diagnoses often experience more persistent and complex sleep problems compared to their typically developing peers [30]. Several longitudinal studies have identified early sleep difficulties as potential risk indicators for diagnosis of later ASDs [18,30]. Specifically, shorter sleep duration, delayed sleep onset and wake times, and frequent midday awakenings have been associated with an increased likelihood of subsequent ASD diagnosis. The timing of sleep-related interventions appears crucial, with recommendations focusing on the period between 3 and 6 months of age, when circadian rhythms become established in the suprachiasmatic nucleus [15,31]. Our findings align with and extend this literature: M-CHAT scores at 12 months showed significant correlations with multiple sleep-related characteristics, including reduced sleep duration, later bedtime, and difficulties with sleep onset (including irritability and prolonged time to fall asleep). These results suggest that infants showing early signs of developmental concerns, as indicated by elevated M-CHAT scores, may already be experiencing sleep-related challenges. Early identification of these sleep patterns could provide opportunities for targeted interventions, potentially benefiting not only the infant’s sleep and neurodevelopmental trajectory but also family functioning and overall cognitive outcomes.

### Relationship Between Inflammatory Markers and Early Developmental Characteristics

Increasing evidence suggests that chronic inflammation plays a role in atypical neurodevelopment, although the underlying mechanisms remain unclear. For example, microglia, crucial for brain maturation and function, are increasingly recognized to play a decisive role in various developmental and cognitive conditions [32]. Microglia produce inflammatory mediators and reactive oxygen species, potentially leading to neuronal degeneration, white matter abnormalities, and decreased neurogenesis observed in conditions such as ASD or schizophrenia [32]. The secretion of signaling molecules and cytokines may promote crosstalk between microglia and astrocytes, leading to endothelial dysfunction and blood-brain barrier permeability impairment [33]. Multiple studies have reported increased concentrations of inflammatory cytokines such as IL-6 and IL-1β and a decrease in anti-inflammatory cytokines (IL-10 and transforming growth factor [TGF]-β) in the peripheral blood and cerebrospinal fluid of individuals with ASD [12,13,33]. The persistent elevation of these inflammatory cytokines may reflect ongoing inflammatory processes [14].

Maternal infections during pregnancy and early childhood are known to influence early development, and changes in cytokine profiles may be shaped by environmental exposures during prenatal and early childhood periods [32]. Typically, the maternal immune system shifts to a more tolerant state during pregnancy, characterized by a decrease in inflammatory cytokines and an increase in regulatory cytokines [12]. However, mothers whose children later receive neurodevelopmental diagnoses have been reported to have significantly higher levels of various inflammatory cytokines and chemokines, such as IL-1α, IL-4, IL-6, and interferon [IFN]-γ in serum during midpregnancy [34,35]. Studies have also reported alterations in melatonin secretion in individuals with ASD, particularly decreased secretion of melatonin and its metabolites at night and altered circadian rhythm [33]. Low maternal melatonin levels during pregnancy have been associated with an increased likelihood of subsequent ASD and intellectual disability diagnoses [36], although there is no consensus on this matter. Contrary to previous research, our study found associations between increased melatonin and decreased IL-1β and M-CHAT scores in midpregnancy. These associations were not observed in maternal blood in late pregnancy. Factors such as the intensity, timing, and duration of maternal inflammatory responses may be crucial for influencing the developing child’s brain. Differences in sample processing and clinical sample variation may also contribute to these findings.

Higher levels of IL-1β in neonatal samples have been associated with an increased likelihood of ASD diagnosis [37]. Moreover, neonatal samples from infants later diagnosed with ASD showed decreased levels of many cytokines (IFN-γ, IL-2, IL-4, IL-6, and IL-10), suggesting reduced immune cell activity in the neonatal period [38]. Interestingly, consistent with previous studies, our analysis found significant correlations between increased IL-1β and decreased IL-6 in cord blood and M-CHAT scores. These results suggest 2 possibilities: first, the cytokine profile in umbilical cord blood samples may predict subsequent developmental characteristics similarly to neonatal samples; second, the M-CHAT assessment at 12 months may effectively identify early developmental concerns before formal diagnostic evaluations. However, these observed relationships between IL-1β and M-CHAT were derived using polychoric or polyserial correlation analysis, and statistical significance might be lost if other statistical methods accounting for outliers were applied. Given that these cytokine data showed very low predictive contribution to the model, and considering that IL-1β is a bioactive substance that can be unstable and challenging to measure in blood in clinical settings, the correlations found in this study should be interpreted with caution.

### Maternal Psychological Well-Being as a Predictor of Early Developmental Characteristics

Focusing on maternal mental and physical status, it has been previously reported that persistent anxiety during pregnancy is significantly associated with ASD in children [8]; further, persistent depression is associated with developmental delay at 18 months [9]. Our study found correlations between K6, EPDS, and M-CHAT, consistent with previous reports. Notably, we identified a significant positive correlation between MIBS and M-CHAT scores at 1 month post partum. While previous studies have shown strong correlations between MIBS and EPDS from early postpartum through 12 months [10], our study is among the first to examine MIBS scores as an independent predictor of early developmental characteristics. Although enhanced parent-child bonding may not directly improve social functioning in children with ASD [39], our findings suggest that MIBS assessment may be valuable for the early identification of developmental concerns.

Our longitudinal analysis demonstrates that early postnatal maternal psychological measures—including emotional state (EPDS), psychological distress (K6), and MIBS—correlate with infant developmental characteristics as assessed by M-CHAT scores. These findings emphasize the importance of comprehensive maternal psychological monitoring in the postpartum period. Even when EPDS scores do not indicate clinical depression, attention to other maternal psychological indicators, particularly MIBS and K6 scores, may provide valuable insights for early developmental screening.

### Limitations

This study has several limitations. First, the pathogeneses of neurodevelopmental disorders are multifactorial; therefore, the presence of unobserved confounding factors cannot be excluded when interpreting the findings of this study. The interrelationship of various sleep and psychological measures (Pittsburgh Sleep Quality Index, K6, MIBS, EPDS, etc) assessed at different time points may introduce potential biases. Parental ratings of child sleep status are inherently subjective and could be influenced by parents’ own psychological stress or sleep issues, potentially affecting the results. Additionally, the collection and measurement of maternal and cord blood biomarkers lack standardized timing protocols and do not account for diurnal variations, necessitating consideration of potential measurement errors in the analysis.

Second, the sample size is small, and the results are preliminary. Future studies should validate these findings using larger samples. Only Japanese participants were included in this study, and no analysis of cultural and social influences due to racial or ethnic differences was conducted. Furthermore, there was considerable variation in the number of mother-child pairs recruited across study sites. While recruitment was not intentionally biased, potential disparities in research resources and physician-patient relationships among facilities may have influenced informed consent rates. Of note is that our longitudinal study may not be fully representative of the Japanese population.

Third, the use of M-CHAT scores at 12 months as an indicator of early developmental characteristics has inherent limitations. While M-CHAT is a validated screening tool, our assessment was based on parent-reported scoring, and due to the short observation period, we were unable to confirm subsequent neurodevelopmental diagnoses. In an ongoing study, we plan to assess ASD using the Kinder Infant Development Scale and Social Responsiveness Scale 2nd edition at 36 months of age.

### Conclusions

Understanding the characteristics of early development and their predictors is essential for improving strategies for early detection and support of neurodevelopmental problems.

Our study demonstrates that a systematic analysis of multiple longitudinal factors can reveal important patterns in early development, particularly in preverbal social behaviors as assessed by M-CHAT scores at 12 months. The results indicate that early developmental characteristics are influenced by several key factors, particularly maternal-infant bonding, maternal anxiety, and infant sleep patterns. The temporal dynamics of these relationships suggest that monitoring specific combinations of factors—including maternal psychological well-being and infant sleep patterns—may enhance our ability to identify early signs of developmental concerns. Future studies with larger and more diverse population samples are needed to validate these findings and establish their generalizability for clinical practice.

## Supplementary material

10.2196/58337 Multimedia Appendix 1 Maternal and infant assessments with questionnaires.

10.2196/58337 Multimedia Appendix 2 Characteristics of participants.

10.2196/58337 Multimedia Appendix 3 Distribution of M-CHAT scores for 12-month-old children aged 12 months. M-CHAT: Modified Checklist for Autism in Toddlers.

10.2196/58337 Multimedia Appendix 4 Correlation between the M-CHAT scores and each variable. M-CHAT:Modified Checklist for Autism in Toddlers.

10.2196/58337 Multimedia Appendix 5 Correlation analysis.

10.2196/58337 Multimedia Appendix 6 Logistic regression model with L2 regularization predicting the DD positive group. DD: developmental diversity.

## Abbreviations

3DSS: 3D Sleep Scale
AQ-J-10: Autism-Spectrum Quotient Japanese short version
ASD: autism spectrum disorder
EPDS: Edinburgh Postnatal Depression Scale
IFN: interferon
IL: interleukin
K6: Kessler Psychological Distress Scale
M-CHAT: Modified Checklist for Autism in Toddlers
MIBS: Mother-to-Infant Bonding Scale
MIBS-J: Japanese version of the Mother-to-Infant Bonding Scale
p.adjust: adjusted *P* value
PSQIG: Pittsburgh Sleep Quality Index global score
SHAP: Shapley Additive Explanations
STROBE: Strengthening the Reporting of Observational Studies in Epidemiology
TGF: transforming growth factor
