# Supplementary material for: Multilevel Factors and Indicators of Atypical Neurodevelopment During Early Infancy in Japan: Prospective, Longitudinal, Observational Study
Source: JMIR Pediatr Parent. 2025 Apr 4;8:e58337. doi: 10.2196/58337 (PMC11990654; doi:10.2196/58337)
Supplement: Multimedia Appendix 1 [file pediatrics-v8-e58337-s001.docx]

## Maternal Assessments With Questionnaires

### Pittsburgh Sleep Quality Index

The PSQI is a self-administered questionnaire widely used to assess quantitative or qualitative sleep problems [1]. It includes seven subscales (sleep quality, latency, duration, habitual sleep efficiency, sleep disturbances, sleep medications, and daytime dysfunction). The PSQI global (PSQIG) scores range from 0 to 21, with PSQIG >5 indicating poor sleep quality. The Japanese version of the PSQI (PSQI-J) has a high overall reliability coefficient (Cronbach’s alpha=0.77), and a PSQI-J global score of 5.5 is the optimal cutoff for sleep quality in patients with mental illness [2].

### 3-Dimensional Sleep Scale

In today's 24-hour society, it has been pointed out that phase problems such as irregular waking and sleeping times may negatively affect health [3]. The PSQI can capture quantitative or qualitative sleep problems but cannot measure or score phase problems. The 3DSS is a self-administered questionnaire that can identify issues with sleep phase, quality, or quantity [4]. It consists of three categories (sleep phase, quality, and quantity), each comprising five items (15 items in total), asking the subject about sleep habits. The questions about the Sleep phase addressed in this study are as follows.

1. Go to bed at a fixed time on weekdays and weekends.
2. I wake up at a fixed time on weekdays and weekends.
3. I have a well-balanced breakfast every day.
4. 'Morningness' is better suited to me than 'eveningness.'
5. What time do you get up on weekdays?

Responses to each item were selected from 1) always, 2) often, 3) rarely, and 4) never. For question 5, the options are 1) approximately 6:00 a.m. or earlier than 6:00 a.m., 2) about 6:30 a.m., 3) approximately 7:00 a.m., and 4) later than 7:00 a.m. Each item is scored on a scale from 0 to 3 (total score 0–15), with higher scores indicating better sleep status. The cutoff value for sleep disturbance is 8/9, with a Cronbach's alpha of 0.685.

### Edinburgh Postnatal Depression Scale (EPDS)

The EPDS was developed as a screening tool for postpartum depression and is now validated and widely used outside the postpartum period [5,6]. It is a 10-item self-administered questionnaire (total score range: 0–30) that assesses a woman's emotional state over the previous seven days. The cutoff value for postpartum depression varies among national language versions, with 8/9 recommended screening for the Japanese version [7].

### Kessler Psychological Distress Scale

The K6 was developed to screen for psychiatric disorders such as mood and anxiety disorders [8] and is widely used as an indicator of the degree of mental health problems, including psychological stress [9–11]. It is a 6-item self-administered questionnaire (rated on a scale of 0–4, with a total score of 0–24) about feelings over the previous 30 days. The optimal cutoffs are 4/5 for mood anxiety disorder and 12/13 for severe mental illness.

### Autism-Spectrum Quotient

The AQ is a self-administered questionnaire developed to measure the extent of autistic traits or their broader phenotype in adults without intellectual disability [12]. It is widely used to clinically screen for ASD and to determine the extent of an individual's disability. The AQ-J-10 consists of the following 10 items:

1. I prefer to do things with others than by myself.
2. Other people often call what I say rude.
3. I tend to have strong interests.
4. When reading a story, I have difficulty understanding the characters' intentions.
5. I would go to the theater rather than a museum.
6. I am often the last to understand the meaning of a joke.
7. I can quickly tell what a person thinks or feels by looking at their face.
8. I like to gather information about categories of things (e.g., cars, birds, trains, types of plants, etc.).
9. It is hard for me to imagine what it would be like to be someone else.
10. It is difficult for me to know what one's intentions are.

Responses are provided using a 4-point scale, with 1 point assigned for selecting two options indicating a high level of autism, while 0 points are assigned for selecting two options indicating a low level of autism (total 0–10 points). The cutoff value for pervasive developmental disorders is 7 points, indicating a high positive predictive value [13].

### Mother-to-Infant Bonding Scale

Screening instruments to assess mothers' attachment formation to their babies during the perinatal period, such as the Mother-Infant Bonding Questionnaire (MIBQ) [14] and its adaptation, the MIBS [15] have been developed.

The MIBS-J was developed based on the MIBQ and has been used to screen for maternal emotional problems with newborns and has shown high reliability and validity [16]. Ten questions are answered using a 4-point scale (total score 0–30), with higher scores indicating more negative feelings toward the baby. The optimal cutoff score for postpartum depression, which increases the risk of neonatal abuse, at one month postpartum, is considered to be 4/5 [17].

## Infant Sleep Status Questionnaires

### At 1 month of age

During the newborn period (up to one month of age), babies sleep between 12 and 16 hours a day, usually between 2 and 4 hours (3 hours on average), with no distinction between day and night.

How was your child’s newborn period? Please indicate how much of the following occurred during the week.

1: never at all, 2: 1–2 times a week, 3: 3–4 times a week, 4: 5–6 times a week, 5: every day.

1. Exhibits a regular pattern of waking up approximately every 3 hours and sleeping more than 10 hours throughout the day.
2. Sleeps well and is easy to manage.
3. Wakes up several times at night, does not sleep, and takes several hands.
4. Takes a long time to fall asleep.
5. Is always irritable and crying.
6. Seems to get extremely limited sleep each day.

### At 6 and 12 months of age

We would like to ask you about your child's sleep and lifestyle. For each of the following items, please choose one from the following options that have applied to your child during the past two weeks.

1. How long does your child sleep at night on average?

1: ≤ 8 hours, 2: 8-9 hours, 3: 9-10 hours, 4: ≥ 10 hours.

1. How long does your child sleep for naps on average?

1: < 1 hour, 2: 1-2 hours, 3: ≥ 2 hours.

1. What time does your child fall asleep on weekdays on average?

1: before 9 p.m., 2: 9 p.m. to 10 p.m., 3: 10 p.m. to 11 p.m., 4: 11 p.m. to 12 p.m., 5: after 12 p.m.

1. What time does your child fall asleep on holidays on average?

1: before 9 p.m., 2: 9 p.m. to 10 p.m., 3: 10 p.m. to 11 p.m., 4: 11 p.m. to 12 p.m., 5: after 12 p.m.

1. What time does your child wake up on weekdays on average?

1: before 7 a.m., 2: 7 a.m. to 8 a.m., 3: 8 a.m. to 9 a.m., 4: after 9 a.m.

1. What time does your child wake up on holiday on average?

1: before 7 a.m., 2: 7 a.m. to 8 a.m., 3: 8 a.m. to 9 a.m., 4: after 9 a.m.

Please indicate how much of the following occurred during the week.

1: never at all, 2: 1-2 times a week, 3: 3-4 times a week, 4: 5-6 times a week, 5: every day.

1. Sleeps well and is easy to manage.
2. Wakes up several times at night, does not sleep, and takes several hands.
3. Takes a long time to fall asleep (does not fall asleep within 1 hour).
4. Takes a long time to fall asleep (fall asleep within 1 hour).
5. Is irritable when falling asleep (severe).
6. Is irritable when falling asleep (minor).
7. Wakes up more than 3 times during the night while crying.
8. Stays awake for more than 1 hour at nocturnal awakening.
9. Is always irritable and crying throughout the day,
10. Feeding every time when the baby cries at night.

## References

1. Buysse DJ, Reynolds CF 3rd, Monk TH, Berman SR, Kupfer DJ. The Pittsburgh Sleep Quality Index: a new instrument for psychiatric practice and research. *Psychiatry Res*. 1989;28(2):193-213.

2. Doi Y, Minowa M, Uchiyama M, et al. Psychometric assessment of subjective sleep quality using the Japanese version of the Pittsburgh Sleep Quality Index (PSQI-J) in psychiatric disordered and control subjects. *Psychiatry Res*. 2000;97(2):165-172.

3. Kitamura S, Hida A, Watanabe M, et al. Evening preference is related to the incidence of depressive states independent of sleep-wake conditions. *Chronobiol Int*. 2010;27(9-10):1797-1812.

4. Matsumoto Y, Uchimura N, Ishida T, et al. Reliability and validity of the 3 Dimensional Sleep Scale (3DSS)--day workers version--in assessing sleep phase, quality, and quantity. *Sangyo Eiseigaku Zasshi*. 2014;56(5):128-140.

5. Cox JL, Holden JM, Sagovsky R. Detection of postnatal depression. Development of the 10-item Edinburgh Postnatal Depression Scale. *Br J Psychiatry*. 1987;150:782-786.

6. Murray D, Cox JL. Screening for depression during pregnancy with the edinburgh depression scale (EDDS). *J Reprod Infant Psychol*. 1990;8(2):99-107.

7. Okano T. Validation and reliability of a Japanese version of the EPDS. *Archives of Psychiatric Diagnosis and Clinical Evaluation*. 1996;7:525-533.

8. Kessler RC, Andrews G, Colpe LJ, et al. Short screening scales to monitor population prevalences and trends in non-specific psychological distress. *Psychol Med*. 2002;32(6):959-976.

9. Sakurai K, Nishi A, Kondo K, Yanagida K, Kawakami N. Screening performance of K6/K10 and other screening instruments for mood and anxiety disorders in Japan. *Psychiatry Clin Neurosci*. 2011;65(5):434-441.

10. Fletcher RJ, Feeman E, Garfield C, Vimpani G. The effects of early paternal depression on children’s development. *Med J Aust*. 2011;195(11-12):685-689.

11. Baggaley RF, Ganaba R, Filippi V, et al. Detecting depression after pregnancy: the validity of the K10 and K6 in Burkina Faso. *Trop Med Int Health*. 2007;12(10):1225-1229.

12. Baron-Cohen S, Wheelwright S, Skinner R, Martin J, Clubley E. The Autism-Spectrum Quotient (AQ): Evidence from Asperger Syndrome/High-Functioning Autism, Malesand Females, Scientists and Mathematicians. *J Autism Dev Disord*. 2001;31(1):5-17.

13. Kurita H, Koyama T, Osada H. Autism-Spectrum Quotient-Japanese version and its short forms for screening normally intelligent persons with pervasive developmental disorders. *Psychiatry Clin Neurosci*. 2005;59(4):490-496.

14. Channi Kumar R. “Anybody’s child”: severe disorders of mother-to-infant bonding. *Br J Psychiatry*. 1997;171(2):175-181.

15. Taylor A, Atkins R, Kumar R, Adams D, Glover V. A new Mother-to-Infant Bonding Scale: links with early maternal mood. *Arch Womens Ment Health*. 2005;8(1):45-51.

16. Yoshida K, Yamashita H, Conroy S, Marks M, Kumar C. A Japanese version of Mother-to-Infant Bonding Scale: factor structure, longitudinal changes and links with maternal mood during the early postnatal period in Japanese mothers. *Arch Womens Ment Health*. 2012;15(5):343-352.

17. Matsunaga A, Takauma F, Tada K, Kitamura T. Discrete category of mother-to-infant bonding disorder and its identification by the Mother-to-Infant Bonding Scale: A study in Japanese mothers of a 1-month-old. *Early Hum Dev*. 2017;111:1-5.
