# Supplementary material for: Multilevel Factors and Indicators of Atypical Neurodevelopment During Early Infancy in Japan: Prospective, Longitudinal, Observational Study
Source: JMIR Pediatr Parent. 2025 Apr 4;8:e58337. doi: 10.2196/58337 (PMC11990654; doi:10.2196/58337)
Supplement: Multimedia Appendix 2 [file pediatrics-v8-e58337-s002.docx]

The characteristics and results for the 144 mother–child pairs included in the final analysis are presented in Multimedia Appendix 2. The characteristics of the participants at mid-gestation (weeks 24-26 weeks), late gestation (weeks 34-38), at birth at 1 month of age, at 6 months of age, and at 12 months of age are shown in Tables S1–S6, respectively.

### Table S1. Characteristics of participants at mid-gestation (weeks 24–26).

| **Characteristics** |  | Longitudinal group |
| --- | --- | --- |
|  | N^a^ | median (IQR) |
| **1,25(OH)_2_D (pg/mL)** | 143 | 117 (97, 134) |
| **25(OD)D (ng/mL)** | 143 | 9.4 (7.2, 11.8) |
| **Melatonin (pg/mL)** | 142 | 17 (12, 25) |
| **IL-17A (pg/mL)** | 141 | 0.34 (0.15, 0.57) |
| **IL-10 (pg/mL)** | 141 | 0.24 (0.19, 0.34) |
| **IL-1β (pg/mL)** | 141 | 0.03 (0.00, 0.05) |
| **IL-6 (pg/mL)** | 141 | 0.45 (0.33, 0.68) |
| **TNF-α (pg/mL)** | 141 | 1.31 (1.01, 1.61) |
| **3DSS (Phase)** | 141 | 10.0 (9.0, 12.0) |
| **PSQIG** | 137 | 5.0 (3.0, 7.0) |
| **EPDS** | 142 | 3.0 (1.3, 6.0) |
| **K6** | 144 | 1.0 (0.0, 4.0) |

^a^Number of participants without missing value.

### Table S2. Characteristics of participants at late gestation (weeks 34–38).

| **Characteristics** |  | Longitudinal group |
| --- | --- | --- |
|  | N^a^ | median (IQR) |
| **1,25(OH)_2_D (pg/mL)** | 137 | 127 (106, 141) |
| **25(OD)D (ng/mL)** | 135 | 8.6 (7.0, 11.1) |
| **Melatonin (pg/mL)** | 134 | 30 (21, 63) |
| **IL-17A (pg/mL)** | 137 | 0.30 (0.15, 0.50) |
| **IL-10 (pg/mL)** | 137 | 0.23 (0.18, 0.34) |
| **IL-1β (pg/mL)** | 137 | 0.03 (0.00, 0.05) |
| **IL-6 (pg/mL)** | 137 | 0.64 (0.45, 0.90) |
| **TNF-α (pg/mL)** | 137 | 1.37 (1.08, 1.79) |
| **3DSS (Phase)** | 138 | 10.0 (7.0, 12.0) |
| **AQ-J-10** | 138 | 2.0 (1.0, 3.0) |
| **PSQIG** | 131 | 6.0 (4.5, 9.0) |
| **EPDS** | 139 | 4.0 (1.5, 7.0) |
| **K6** | 139 | 2.0 (0.0, 4.0) |

^a^Number of participants without missing value.

### Table S3. Characteristics of participants at birth.

| **Characteristics** |  | Longitudinal group |
| --- | --- | --- |
|  | N^a^ | median (IQR) |
| **1,25(OH)_2_D (pg/mL)** | 130 | 56 (44, 69) |
| **25(OD)D (ng/mL)** | 133 | 9.6 (7.6, 12.0) |
| **Melatonin (pg/mL)** | 119 | 30 (20, 59) |
| **IL-17A (pg/mL)** | 127 | 0.00 (0.00, 0.17) |
| **IL-10 (pg/mL)** | 127 | 0.44 (0.30, 0.74) |
| **IL-1β (pg/mL)** | 127 | 0.06 (0.02, 0.23) |
| **IL-6 (pg/mL)** | 127 | 1 (1, 3) |
| **TNF-α (pg/mL)** | 127 | 2.4 (1.6, 3.1) |

^a^Number of participants without missing value.

### Table S4. Characteristics of participants at 1 month of age.

| **Characteristics** |  | Longitudinal group |
| --- | --- | --- |
|  | N^a^ | number (%) |
| **Regular sleep–wake cycle, n (%)** | 137 |  |
| Never |  | 9 (6.6%) |
| 1–2 times per week |  | 11 (8.0%) |
| 3–4 times per week |  | 16 (12%) |
| 5–6 times per week |  | 28 (20%) |
| Everyday |  | 73 (53%) |
| **Sleeps well and is easy to manage, n (%)** | 136 |  |
| Never |  | 22 (16%) |
| 1–2 times per week |  | 39 (29%) |
| 3–4 times per week |  | 36 (26%) |
| 5–6 times per week |  | 26 (19%) |
| Everyday |  | 13 (9.6%) |
| **Wakes up several times, fussy, n (%)** | 136 |  |
| Never |  | 38 (28%) |
| 1–2 times per week |  | 64 (47%) |
| 3–4 times per week |  | 21 (15%) |
| 5–6 times per week |  | 8 (5.9%) |
| Everyday |  | 5 (3.7%) |
| **Takes a long time to fall asleep, n (%)** | 136 |  |
| Never |  | 31 (23%) |
| 1–2 times per week |  | 67 (49%) |
| 3–4 times per week |  | 19 (14%) |
| 5–6 times per week |  | 13 (9.6%) |
| Everyday |  | 6 (4.4%) |
| **Always irritable and crying, n (%)** | 136 |  |
| Never |  | 62 (46%) |
| 1–2 times per week |  | 53 (39%) |
| 3–4 times per week |  | 14 (10%) |
| 5–6 times per week |  | 4 (2.9%) |
| Everyday |  | 3 (2.2%) |
| **Short sleep duration, n (%)** | 136 |  |
| Never |  | 90 (66%) |
| 1–2 times per week |  | 30 (22%) |
| 3–4 times per week |  | 11 (8.1%) |
| 5–6 times per week |  | 0 (0.0%) |
| Everyday |  | 5 (3.7%) |
| **PSQIG** | 140 | 8.0 (6.0, 10.0)^b^ |
| **EPDS** | 141 | 2.0 (1.0, 5.0)^b^ |
| **K6** | 142 | 1.0 (0.0, 3.0)^b^ |
| **MIBS-J** | 142 | 1.0 (0.0, 3.0)^b^ |

^a^Number of participants without missing value.

^b^median (IQR)

### Table S5. Characteristics of participants at 6 months of age.

| **Characteristics** |  | Longitudinal group |
| --- | --- | --- |
|  | N^a^ | number (%) |
| **Nighttime sleeping hours** | 139 |  |
| ≤ 8 hours |  | 20 (14%) |
| 8– 9 hours |  | 44 (32%) |
| 9–10 hours |  | 58 (42%) |
| ≥ 10 hours |  | 17 (12%) |
| **Naptime sleeping hours** | 138 |  |
| < 1 hour |  | 11 (8.0%) |
| 1–2 hours |  | 88 (64%) |
| ≥ 2 hours |  | 39 (28%) |
| **Weekday bedtime** | 139 |  |
| < 9 pm |  | 44 (32%) |
| 9–10 pm |  | 65 (47%) |
| 10–11 pm |  | 18 (13%) |
| 11–12 pm |  | 11 (7.9%) |
| > 12 pm |  | 1 (0.7%) |
| **Holiday bedtime** | 139 |  |
| < 9 pm |  | 41 (29%) |
| 9–10 pm |  | 69 (50%) |
| 10–11 pm |  | 15 (11%) |
| 11–12 pm |  | 13 (9.4%) |
| > 12 pm |  | 1 (0.7%) |
| **Weekday wake-up time** | 139 |  |
| < 7 am |  | 56 (40%) |
| 7–8 am |  | 61 (44%) |
| 8–9 am |  | 19 (14%) |
| > 9 pm |  | 3 (2.2%) |
| **Holiday wake-up time** | 139 |  |
| < 7 am |  | 46 (33%) |
| 7–8 am |  | 66 (47%) |
| 8–9 am |  | 24 (17%) |
| > 9 pm |  | 3 (2.2%) |
| **Sleeps well and is easy to manage** | 139 |  |
| Never |  | 19 (14%) |
| 1–2 times per week |  | 34 (24%) |
| 3–4 times per week |  | 22 (16%) |
| 5–6 times per week |  | 30 (22%) |
| Everyday |  | 34 (24%) |
| **Wakes up several times, fussy** | 138 |  |
| Never |  | 75 (54%) |
| 1–2 times per week |  | 39 (28%) |
| 3–4 times per week |  | 9 (6.5%) |
| 5–6 times per week |  | 6 (4.3%) |
| Everyday |  | 9 (6.5%) |
| **Takes a long time (≥ 1 hour) to fall asleep** | 137 |  |
| Never |  | 80 (58%) |
| 1–2 times per week |  | 43 (31%) |
| 3–4 times per week |  | 12 (8.8%) |
| 5–6 times per week |  | 2 (1.5%) |
| Everyday |  | 0 (0.0%) |
| **Takes a long time (< 1 hour) to fall asleep** | 137 |  |
| Never |  | 41 (30%) |
| 1–2 times per week |  | 39 (28%) |
| 3–4 times per week |  | 21 (15%) |
| 5–6 times per week |  | 17 (12%) |
| Everyday |  | 19 (14%) |
| **irritable when falling asleep (severe)** | 138 |  |
| Never |  | 83 (60%) |
| 1–2 times per week |  | 29 (21%) |
| 3–4 times per week |  | 14 (10%) |
| 5–6 times per week |  | 8 (5.8%) |
| Everyday |  | 4 (2.9%) |
| **irritable when falling asleep (minor)** | 136 |  |
| Never |  | 32 (24%) |
| 1–2 times per week |  | 60 (44%) |
| 3–4 times per week |  | 23 (17%) |
| 5–6 times per week |  | 8 (5.9%) |
| Everyday |  | 13 (9.6%) |
| **Wakes up ≥ 3 times during the night** | 138 |  |
| Never |  | 93 (67%) |
| 1–2 times per week |  | 30 (22%) |
| 3–4 times per week |  | 4 (2.9%) |
| 5–6 times per week |  | 6 (4.3%) |
| Everyday |  | 5 (3.6%) |
| **Staying awake (≥ 1 hour) at nocturnal awakening** | 138 |  |
| Never |  | 111 (80%) |
| 1–2 times per week |  | 25 (18%) |
| 3–4 times per week |  | 2 (1.4%) |
| 5–6 times per week |  | 0 (0.0%) |
| Everyday |  | 0 (0.0%) |
| **Always irritable and crying** | 138 |  |
| Never |  | 95 (69%) |
| 1–2 times per week |  | 36 (26%) |
| 3–4 times per week |  | 6 (4.3%) |
| 5–6 times per week |  | 0 (0.0%) |
| Everyday |  | 1 (0.7%) |
| **Feeding every time when the baby cries at night** | 138 |  |
| Never |  | 66 (48%) |
| 1–2 times per week |  | 21 (15%) |
| 3–4 times per week |  | 5 (3.6%) |
| 5–6 times per week |  | 10 (7.2%) |
| Everyday |  | 36 (26%) |

^a^Number of participants without missing value.

### Table S6. Characteristics of participants at 12 months of age.

| **Characteristics** |  | Longitudinal group |
| --- | --- | --- |
|  | N^a^ | number (%) |
| **Nighttime sleeping hours** | 143 |  |
| ≤ 8 hours |  | 7 (4.9%) |
| 8– 9 hours |  | 50 (35%) |
| 9–10 hours |  | 68 (48%) |
| ≥ 10 hours |  | 18 (13%) |
| **Naptime sleeping hours** | 143 |  |
| < 1 hour |  | 7 (4.9%) |
| 1–2 hours |  | 98 (69%) |
| ≥ 2 hours |  | 38 (27%) |
| **Weekday bedtime** | 143 |  |
| < 9 pm |  | 52 (36%) |
| 9–10 pm |  | 62 (43%) |
| 10–11 pm |  | 21 (15%) |
| 11–12 pm |  | 7 (4.9%) |
| > 12 pm |  | 1 (0.7%) |
| **Holiday bedtime** | 143 |  |
| < 9 pm |  | 45 (31%) |
| 9–10 pm |  | 69 (48%) |
| 10–11 pm |  | 17 (12%) |
| 11–12 pm |  | 10 (7.0%) |
| > 12 pm |  | 2 (1.4%) |
| **Weekday wake-up time** | 143 |  |
| < 7 am |  | 71 (50%) |
| 7–8 am |  | 58 (41%) |
| 8–9 am |  | 12 (8.4%) |
| > 9 pm |  | 2 (1.4%) |
| **Holiday wake-up time** | 143 |  |
| < 7 am |  | 54 (38%) |
| 7–8 am |  | 67 (47%) |
| 8–9 am |  | 17 (12%) |
| > 9 pm |  | 5 (3.5%) |
| **Sleeps well and is easy to manage** | 143 |  |
| Never |  | 28 (20%) |
| 1–2 times per week |  | 21 (15%) |
| 3–4 times per week |  | 23 (16%) |
| 5–6 times per week |  | 33 (23%) |
| Everyday |  | 38 (27%) |
| **Wakes up several times, fussy** | 143 |  |
| Never |  | 60 (42%) |
| 1–2 times per week |  | 44 (31%) |
| 3–4 times per week |  | 13 (9.1%) |
| 5–6 times per week |  | 8 (5.6%) |
| Everyday |  | 18 (13%) |
| **Takes a long time (≥ 1 hour) to fall asleep** | 144 |  |
| Never |  | 89 (62%) |
| 1–2 times per week |  | 37 (26%) |
| 3–4 times per week |  | 14 (9.7%) |
| 5–6 times per week |  | 4 (2.8%) |
| Everyday |  | 0 (0.0%) |
| **Takes a long time (< 1 hour) to fall asleep** | 142 |  |
| Never |  | 38 (27%) |
| 1–2 times per week |  | 44 (31%) |
| 3–4 times per week |  | 26 (18%) |
| 5–6 times per week |  | 12 (8.5%) |
| Everyday |  | 22 (15%) |
| **irritable when falling asleep (severe)** | 142 |  |
| Never |  | 91 (64%) |
| 1–2 times per week |  | 32 (23%) |
| 3–4 times per week |  | 10 (7.0%) |
| 5–6 times per week |  | 4 (2.8%) |
| Everyday |  | 5 (3.5%) |
| **irritable when falling asleep (minor)** | 144 |  |
| Never |  | 48 (33%) |
| 1–2 times per week |  | 57 (40%) |
| 3–4 times per week |  | 20 (14%) |
| 5–6 times per week |  | 6 (4.2%) |
| Everyday |  | 13 (9.0%) |
| **Wakes up ≥ 3 times during the night** | 143 |  |
| Never |  | 88 (62%) |
| 1–2 times per week |  | 31 (22%) |
| 3–4 times per week |  | 7 (4.9%) |
| 5–6 times per week |  | 8 (5.6%) |
| Everyday |  | 9 (6.3%) |
| **Staying awake (≥ 1 hour) at nocturnal awakening** | 144 |  |
| Never |  | 118 (82%) |
| 1–2 times per week |  | 22 (15%) |
| 3–4 times per week |  | 4 (2.8%) |
| 5–6 times per week |  | 0 (0.0%) |
| Everyday |  | 0 (0.0%) |
| **Always irritable and crying** | 144 |  |
| Never |  | 122 (85%) |
| 1–2 times per week |  | 18 (12%) |
| 3–4 times per week |  | 2 (1.4%) |
| 5–6 times per week |  | 1 (0.7%) |
| Everyday |  | 1 (0.7%) |
| **Feeding every time when the baby cries at night** | 144 |  |
| Never |  | 91 (63%) |
| 1–2 times per week |  | 12 (8.3%) |
| 3–4 times per week |  | 6 (4.2%) |
| 5–6 times per week |  | 6 (4.2%) |
| Everyday |  | 29 (20%) |
| **M-CHAT score** | 144 |  |
| 0 |  | 105 (73%) |
| 1 |  | 30 (21%) |
| 2 |  | 5 (3.5%) |
| 3 |  | 3 (2.1%) |
| 4 |  | 1 (0.7%) |

^a^Number of participants without missing value.
