# Supplementary material for: Multilevel Factors and Indicators of Atypical Neurodevelopment During Early Infancy in Japan: Prospective, Longitudinal, Observational Study
Source: JMIR Pediatr Parent. 2025 Apr 4;8:e58337. doi: 10.2196/58337 (PMC11990654; doi:10.2196/58337)
Supplement: Multimedia Appendix 3 [file pediatrics-v8-e58337-s003.docx]

Achievement rates for each preverbal social behavior item of the M-CHAT used in this study are shown in Table S1. Data from a previous report [^1^] on a healthy population are also included. Achievement rates for each preverbal social behavior item of the M-CHAT used in this study are shown in Table S1. The distribution of the M-CHAT scores for the 144 12-month-old children is shown in Figure S1. Figure S2 illustrates the preverbal social behavior item distribution for the 39 children who scored at least 1 on the M-CHAT.

### Table S1. The passage rate of preverbal social behaviors at 12-months of age (%).

| Item no. | Item content | Our cases | Inada et al. [^1^] | | |
| --- | --- | --- | --- | --- | --- |
|  |  | 12 months | 11 months | 12 months | 13 months |
| 1 | Enjoying being swung | 98.6 | 100 | 100 | 100 |
| 2 | Interest in other children | 98.0 | 97.3 | 100 | 97.0 |
| 4 | Enjoying peek-a-boo | 98.6 | 94.6 | 100 | 100 |
| 10 | Eye contact | 95.1 | 100 | 96.6 | 90.9 |
| 12 | Response to smile | 99.3 | 100 | 100 | 100 |
| 14 | Response to name | 97.9 | 100 | 96.6 | 97.0 |
| 5 | Pretend play | 50.3 | 48.6 | 79.3 | 69.7 |
| 6 | Imperative pointing | 58.0 | 43.2 | 75.9 | 87.9 |
| 7 | Declarative pointing | 58.7 | 51.4 | 79.3 | 75.8 |
| 13 | Imitation of acts | 89.6 | 81.1 | 82.8 | 81.8 |
| 15 | Point following | 86.7 | 67.6 | 86.2 | 78.8 |
| 19 | Attracting parent’s attention | 79.9 | 75.7 | 86.2 | 72.7 |
| 8 | Functional play | 45.5 | 27.0 | 41.4 | 21.2 |
| 9 | Bringing object to show | 42.0 | 29.7 | 44.8 | 48.5 |
| 17 | Gaze following | 74.8 | 48.6 | 69.0 | 66.7 |
| 23 | Social reference | 68.5 | 70.3 | 65.5 | 66.7 |

### Figure S1. Distribution of the M-CHAT scores at 12 months of age.


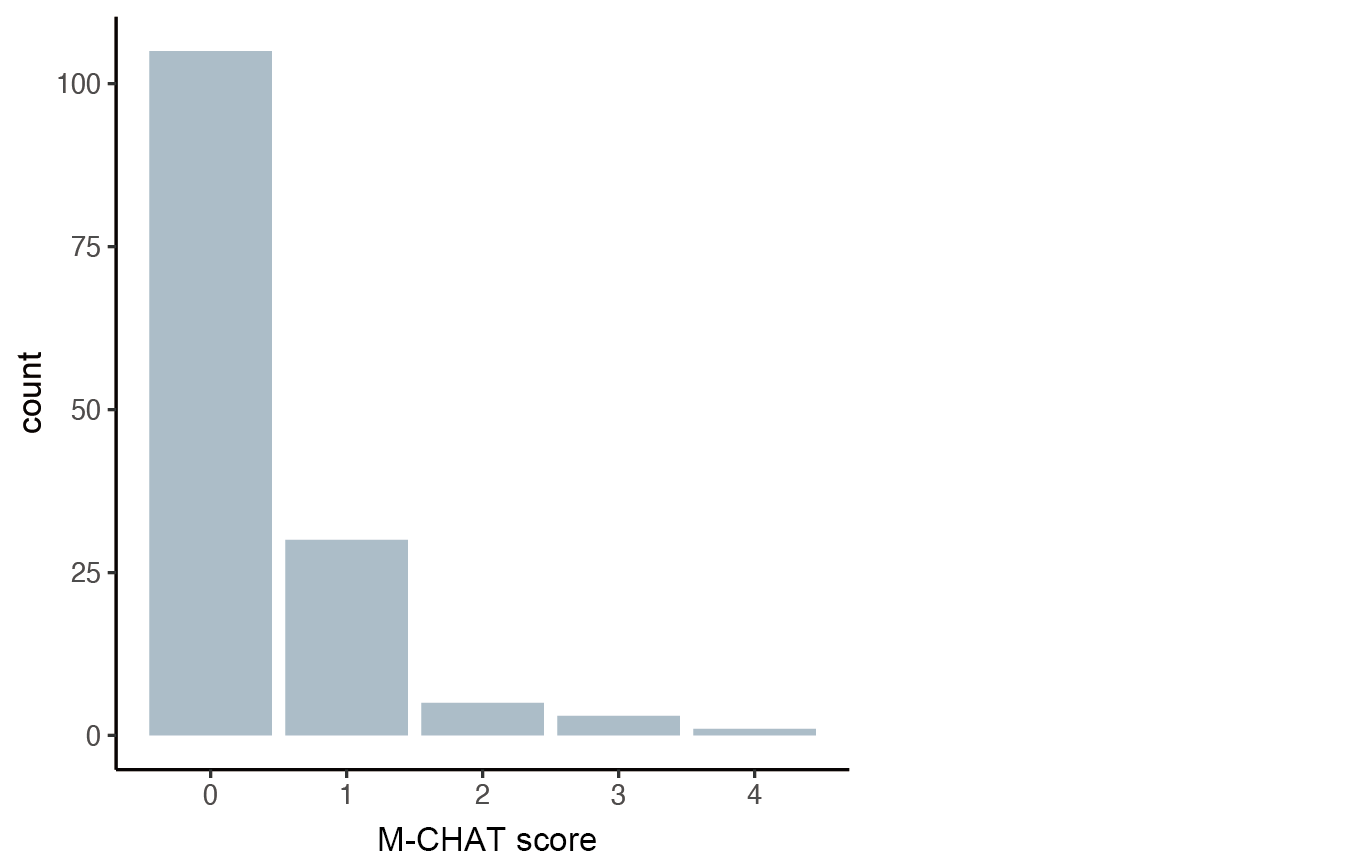


### Figure S2. Relationship between the M-CHAT scores (1–4) and questions. M-CHAT: Modified Checklist for Autism in Toddlers.


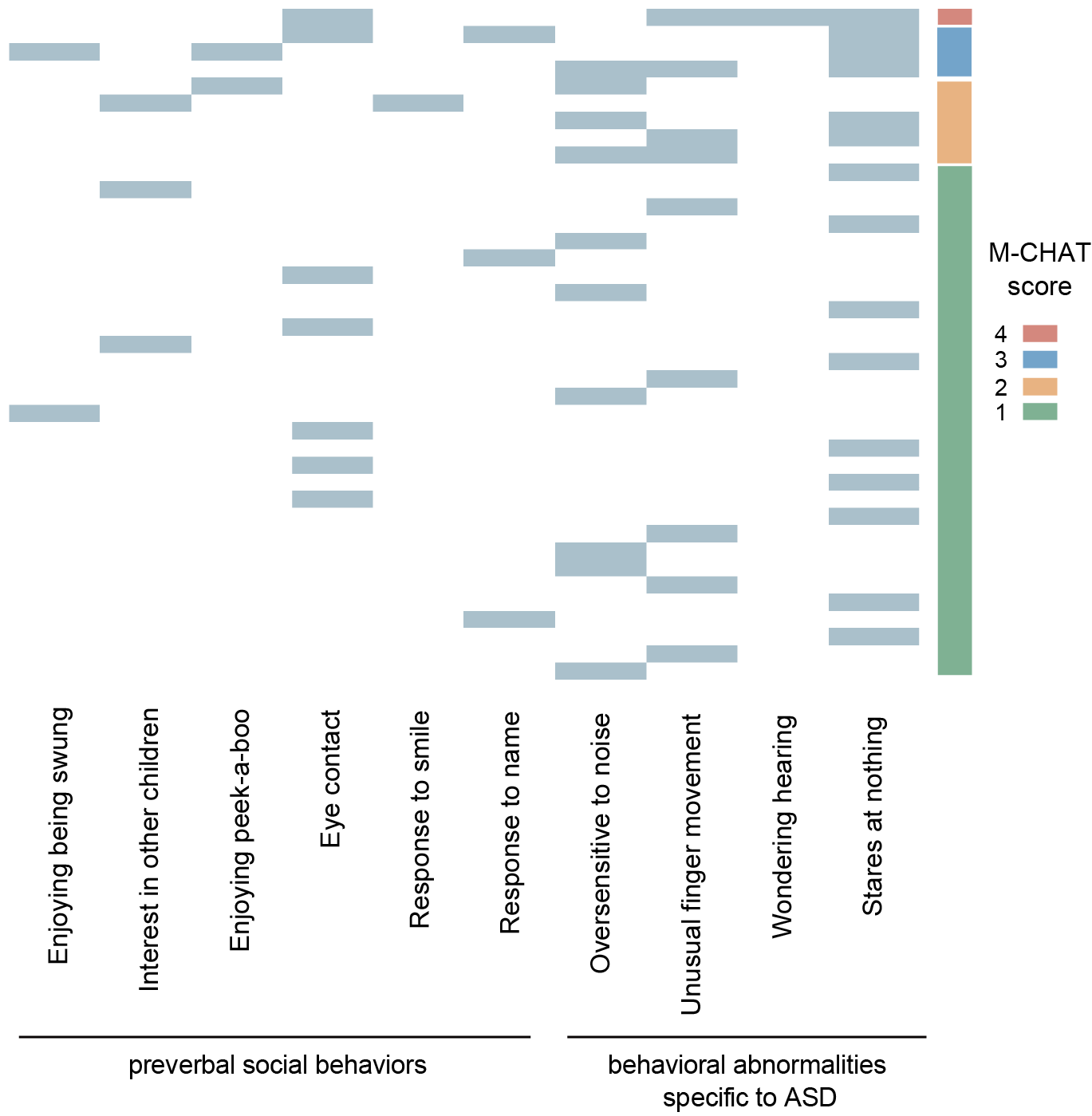


Of the ten items, six from the left are preverbal social behaviors with an extremely high pass rate at 12 months. The four items from the right are behavioral abnormalities specific to ASD—irrespective of the time of appearance.

## Reference

1. Inada N, Kamio Y, Koyama T. Developmental chronology of preverbal social behaviors in infancy using the M-CHAT: Baseline for early detection of atypical social development. *Res Autism Spectr Disord*. 2010;4(4):605-611.
