# Supplementary material for: Multilevel Factors and Indicators of Atypical Neurodevelopment During Early Infancy in Japan: Prospective, Longitudinal, Observational Study
Source: JMIR Pediatr Parent. 2025 Apr 4;8:e58337. doi: 10.2196/58337 (PMC11990654; doi:10.2196/58337)
Supplement: Multimedia Appendix 4 [file pediatrics-v8-e58337-s004.docx]

Table S1 details the correlation coefficients and adjusted p-values between M-CHAT scores and each variable. Each variable is sorted by observation period.

### Table S1. Correlation between M-CHAT scores and each variable. M-CHAT: Modified Checklist for Autism in Toddlers.

| **Observation period** | **Variable** | **Correlation coefficient** | **p-value** | **p.adjust** |
| --- | --- | --- | --- | --- |
| **Baseline** | Maternal age | -0.053 | 0.613 | 0.736 |
|  | Infant sex | -0.125 | 0.355 | 0.623 |
|  | Smoking status | -0.917 | > 0.999 | > 0.999 |
|  | Maternal education | 0.233 | 0.018 | 0.110 |
|  | Paternal education | 0.087 | 0.415 | 0.623 |
|  | Annual household income | -0.087 | 0.329 | 0.623 |
| **Mid-gestation** | 1,25(OH)2D | -0.073 | 0.403 | 0.591 |
|  | 25(OD)D | 0.018 | 0.847 | 0.847 |
|  | Melatonin | 0.270 | < 0.001 | 0.001 |
|  | IL-17A | 0.092 | 0.258 | 0.516 |
|  | IL-10 | -0.327 | 0.030 | 0.090 |
|  | IL-1β | -0.278 | < 0.001 | < 0.001 |
|  | IL-6 | -0.068 | 0.466 | 0.591 |
|  | TNF-α | -0.246 | 0.163 | 0.391 |
|  | 3DSS_Phase | -0.221 | 0.027 | 0.090 |
|  | PSQIG | 0.082 | 0.448 | 0.591 |
|  | EPDS | 0.023 | 0.818 | 0.847 |
|  | K6 | 0.068 | 0.492 | 0.591 |
| **Late gestation** | 1,25(OH)2D | -0.032 | 0.702 | 0.921 |
|  | 25(OD)D | 0.017 | 0.862 | 0.921 |
|  | Melatonin | 0.025 | 0.830 | 0.921 |
|  | IL-17A | -0.051 | 0.716 | 0.921 |
|  | IL-10 | -0.132 | 0.066 | 0.228 |
|  | IL-1β | -0.234 | 0.143 | 0.333 |
|  | IL-6 | -0.006 | 0.921 | 0.921 |
|  | TNF-α | -0.254 | 0.214 | 0.397 |
|  | 3DSS_Phase | -0.145 | 0.154 | 0.333 |
|  | AQ-J-10 | 0.118 | 0.312 | 0.508 |
|  | PSQIG | 0.138 | 0.07 | 0.228 |
|  | EPDS | 0.232 | 0.004 | 0.037 |
|  | K6 | 0.288 | 0.006 | 0.037 |
| **At birth** | 1,25(OH)2D | -0.001 | 0.990 | 0.990 |
|  | 25(OD)D | 0.059 | 0.512 | 0.903 |
|  | Melatonin | 0.085 | 0.519 | 0.903 |
|  | IL-17A | 0.059 | 0.556 | 0.903 |
|  | IL-10 | 0.183 | < 0.001 | < 0.001 |
|  | IL-1β | 0.233 | < 0.001 | < 0.001 |
|  | IL-6 | -0.362 | < 0.001 | < 0.001 |
|  | TNF-α | -0.046 | 0.837 | 0.907 |
|  | Gestational age at birth | -0.067 | 0.719 | 0.907 |
|  | Birth weight | -0.055 | 0.704 | 0.907 |
|  | Apgar scores at 5 min | -0.179 | 0.182 | 0.593 |
|  | Umbilical artery blood pH | -0.132 | 0.289 | 0.750 |
|  | Type of delivery | 0.028 | 0.819 | 0.907 |
| **At 1 month of age** | Regular sleep-wake cycle | -0.010 | 0.923 | 0.923 |
|  | Sleeps well, easy to manage | -0.080 | 0.427 | 0.534 |
|  | Wake several times, fussy | 0.256 | 0.006 | 0.014 |
|  | Take long to fall asleep | 0.247 | 0.005 | 0.014 |
|  | Always irritable, crying | 0.203 | 0.010 | 0.019 |
|  | Short sleep duration | 0.319 | 0.004 | 0.014 |
|  | PSQIG | -0.048 | 0.654 | 0.727 |
|  | EPDS | 0.164 | 0.107 | 0.153 |
|  | K6 | 0.236 | 0.020 | 0.033 |
|  | MIBS-J | 0.320 | 0.001 | 0.009 |
| **At 6 month of age** | Night-time sleeping hours | -0.391 | < 0.001 | < 0.001 |
|  | Nap-time sleeping hours | -0.298 | 0.006 | 0.014 |
|  | Weekday bedtime hour | 0.309 | < 0.001 | < 0.001 |
|  | Holiday bedtime hour | 0.330 | < 0.001 | < 0.001 |
|  | Weekday awakening hour | 0.038 | 0.703 | 0.803 |
|  | Holiday awakening hour | 0.006 | 0.953 | 0.953 |
|  | Sleeps well, easy to manage | -0.131 | 0.249 | 0.443 |
|  | Wake several times, fussy | 0.080 | 0.486 | 0.648 |
|  | Take a long time to fall asleep, >= 1 hour | 0.410 | < 0.001 | < 0.001 |
|  | Take a long time to fall asleep, < 1 hour | 0.064 | 0.581 | 0.715 |
|  | Very irritable when falling asleep | 0.169 | 0.134 | 0.269 |
|  | Slightly irritable when falling asleep | 0.362 | < 0.001 | < 0.001 |
|  | Waking up >= 3 times during the night | 0.023 | 0.868 | 0.926 |
|  | Staying awake for >= 1 hour at mid-wake | 0.266 | 0.004 | 0.010 |
|  | Always irritable, crying | 0.106 | 0.400 | 0.582 |
|  | Feeding every time when baby cries at night | -0.104 | 0.366 | 0.582 |
| **At 12 month of age** | Regular sleep-wake cycle | -0.010 | 0.923 | 0.923 |
|  | Sleeps well, easy to manage | -0.080 | 0.427 | 0.534 |
|  | Wake several times, fussy | 0.256 | 0.006 | 0.014 |
|  | Take long to fall asleep | 0.247 | 0.005 | 0.014 |
|  | Always irritable, crying | 0.203 | 0.010 | 0.019 |
|  | Short sleep duration | 0.319 | 0.004 | 0.014 |
|  | PSQIG | -0.048 | 0.654 | 0.727 |
|  | EPDS | 0.164 | 0.107 | 0.153 |
|  | K6 | 0.236 | 0.020 | 0.033 |
|  | MIBS-J | 0.320 | 0.001 | 0.009 |
