# Supplementary material for: Multilevel Factors and Indicators of Atypical Neurodevelopment During Early Infancy in Japan: Prospective, Longitudinal, Observational Study
Source: JMIR Pediatr Parent. 2025 Apr 4;8:e58337. doi: 10.2196/58337 (PMC11990654; doi:10.2196/58337)
Supplement: Multimedia Appendix 5 [file pediatrics-v8-e58337-s005.docx]

Correlation analysis was performed to determine the interrelationships among the factors affecting infant developmental diversity. Figure S1 shows the correlation network diagram. Table S1 details the correlation coefficients and adjusted p-values among the factors.

### Figure S1. Correlation network diagram between the M-CHAT scores and each correlated variable. M-CHAT: Modified Checklist for Autism in Toddlers.

**
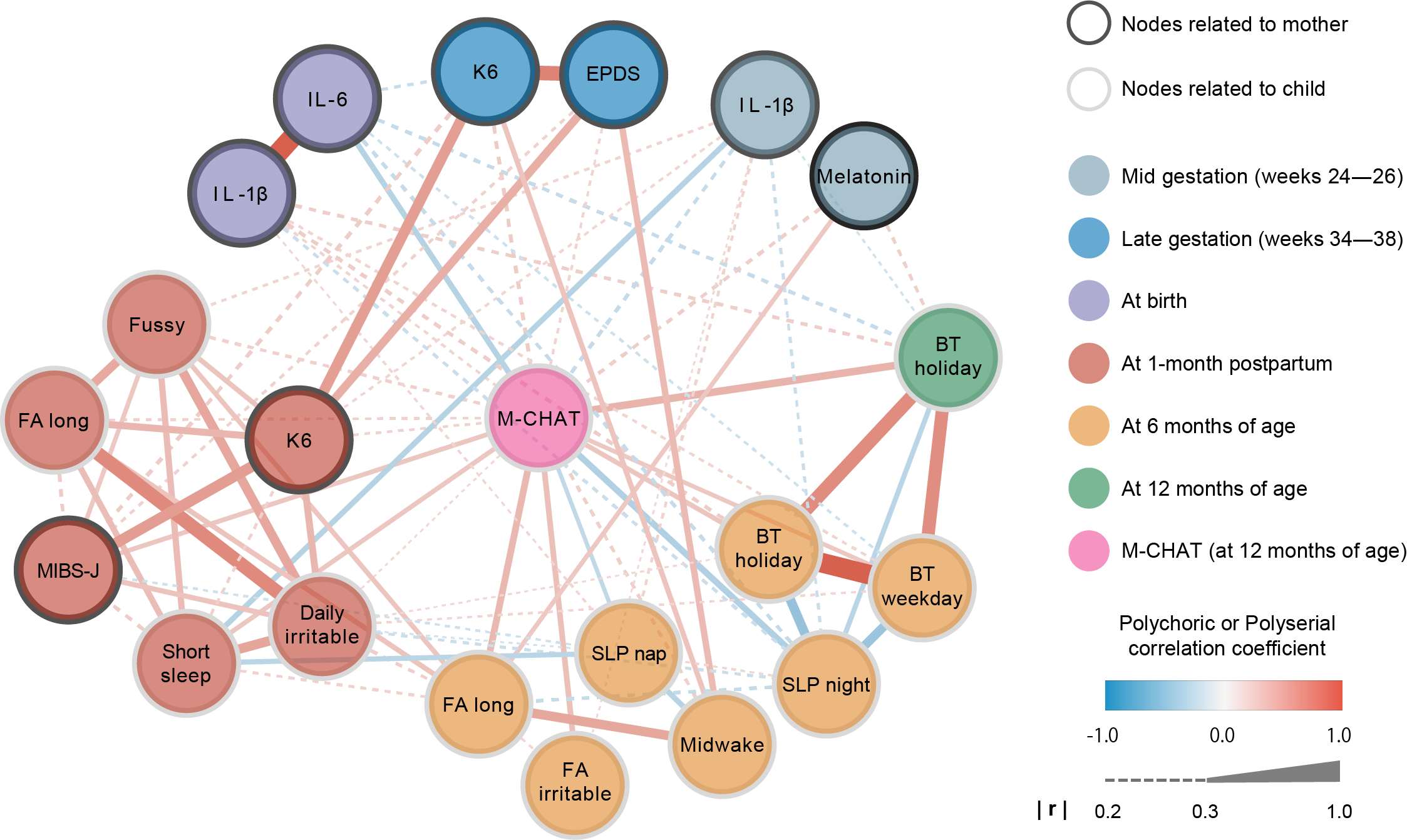
**

Note. Color scale: red, high correlation; blue, low correlation. The width of the edges is related to the absolute value of the correlation coefficient, with .2 ≤ |r| < .3 indicated by a dotted line and |r| ≥ .3 by a solid line. The structure contained 21 nodes and 82 edges. Fussy = wake several times, fussy; FA long = takes long to fall asleep; Daily irritable = is always irritable and crying; Short sleep = short sleep duration; BT = bedtime; SLP = sleeping hours; Midwake = staying awake for more than 1 hour at nocturnal awakening; FA irritable = is slightly irritable when falling asleep.

### Table S1. The interrelationships among the factors affecting infant developmental diversity.

| **Variables** | **Variables** | **Correlation coefficient** | **p-value** | **p.adjust** |
| --- | --- | --- | --- | --- |
| SLP night_6 | SLP nap_6 | ^†^ 0.215 | 0.012 | 0.027 |
| SLP night_6 | Midwake_6 | ^†^ -0.183 | 0.053 | 0.092 |
| SLP night_6 | M-CHAT | ^†^ -0.391 | < 0.001 | < 0.001 |
| SLP night_6 | FA long_6 | ^†^ -0.282 | < 0.001 | 0.001 |
| SLP night_6 | FA irritable_6 | ^†^ -0.189 | 0.036 | 0.068 |
| SLP night_6 | BT weekday_6 | ^†^ -0.509 | < 0.001 | < 0.001 |
| SLP night_6 | BT holiday_6 | ^†^ -0.528 | < 0.001 | < 0.001 |
| SLP night_6 | BT holiday_12 | ^†^ -0.337 | < 0.001 | < 0.001 |
| SLP nap_6 | Midwake_6 | ^†^ -0.354 | 0.007 | 0.017 |
| SLP nap_6 | M-CHAT | ^†^ -0.298 | 0.006 | 0.015 |
| SLP nap_6 | FA long_6 | ^†^ -0.117 | 0.201 | 0.275 |
| SLP nap_6 | FA irritable_6 | ^†^ -0.036 | 0.694 | 0.760 |
| SLP nap_6 | BT weekday_6 | ^†^ -0.146 | 0.086 | 0.140 |
| SLP nap_6 | BT holiday_6 | ^†^ -0.137 | 0.105 | 0.164 |
| SLP nap_6 | BT holiday_12 | ^†^ -0.046 | 0.691 | 0.760 |
| Short sleep_1 | SLP night_6 | ^†^ -0.079 | 0.445 | 0.527 |
| Short sleep_1 | SLP nap_6 | ^†^ -0.340 | 0.002 | 0.005 |
| Short sleep_1 | Midwake_6 | ^†^ 0.150 | 0.285 | 0.368 |
| Short sleep_1 | MIBS-J_1 | ^†^ 0.283 | 0.001 | 0.003 |
| Short sleep_1 | M-CHAT | ^†^ 0.319 | 0.004 | 0.010 |
| Short sleep_1 | K6_1 | ^†^ 0.245 | 0.003 | 0.008 |
| Short sleep_1 | FA long_6 | ^†^ 0.247 | 0.013 | 0.028 |
| Short sleep_1 | FA irritable_6 | ^†^ 0.075 | 0.429 | 0.519 |
| Short sleep_1 | BT weekday_6 | ^†^ 0.204 | 0.039 | 0.073 |
| Short sleep_1 | BT holiday_6 | ^†^ 0.196 | 0.047 | 0.084 |
| Short sleep_1 | BT holiday_12 | ^†^ 0.131 | 0.193 | 0.268 |
| Midwake_6 | M-CHAT | ^†^ 0.266 | 0.004 | 0.010 |
| Midwake_6 | BT holiday_12 | ^†^ -0.060 | 0.651 | 0.728 |
| MIBS-J_1 | SLP night_6 | ^†^ -0.019 | 0.806 | 0.862 |
| MIBS-J_1 | SLP nap_6 | ^†^ -0.225 | 0.004 | 0.010 |
| MIBS-J_1 | Midwake_6 | ^†^ 0.078 | 0.436 | 0.521 |
| MIBS-J_1 | M-CHAT | ^†^ 0.320 | 0.001 | 0.003 |
| MIBS-J_1 | FA long_6 | ^†^ 0.112 | 0.179 | 0.253 |
| MIBS-J_1 | FA irritable_6 | ^†^ 0.078 | 0.312 | 0.396 |
| MIBS-J_1 | BT weekday_6 | ^†^ 0.155 | 0.045 | 0.081 |
| MIBS-J_1 | BT holiday_6 | ^†^ 0.131 | 0.090 | 0.143 |
| MIBS-J_1 | BT holiday_12 | ^†^ 0.115 | 0.241 | 0.318 |
| Melatonin_mid | SLP night_6 | ^‡^ -0.096 | 0.194 | 0.268 |
| Melatonin_mid | SLP nap_6 | ^‡^ 0.005 | 0.950 | 0.975 |
| Melatonin_mid | Short sleep_1 | ^‡^ 0.091 | 0.115 | 0.175 |
| Melatonin_mid | Midwake_6 | ^‡^ 0.037 | 0.652 | 0.728 |
| Melatonin_mid | MIBS-J_1 | ^‡^ 0.096 | 0.016 | 0.034 |
| Melatonin_mid | M-CHAT | ^‡^ 0.270 | < 0.001 | < 0.001 |
| Melatonin_mid | K6_late | ^‡^ 0.009 | 0.929 | 0.962 |
| Melatonin_mid | K6_1 | ^‡^ -0.023 | 0.709 | 0.772 |
| Melatonin_mid | IL-6_birth | ^*^ 0.161 | < 0.001 | < 0.001 |
| Melatonin_mid | IL-1β_mid | ^*^ 0.014 | 0.887 | 0.928 |
| Melatonin_mid | IL-1β_birth | ^*^ 0.088 | 0.012 | 0.025 |
| Melatonin_mid | Fuzzy_1 | ^‡^ 0.098 | 0.227 | 0.305 |
| Melatonin_mid | FA long_6 | ^‡^ 0.317 | < 0.001 | < 0.001 |
| Melatonin_mid | FA long_1 | ^‡^ 0.142 | 0.025 | 0.05 |
| Melatonin_mid | FA irritable_6 | ^‡^ -0.037 | 0.275 | 0.357 |
| Melatonin_mid | EPDS_late | ^‡^ 0.021 | 0.802 | 0.862 |
| Melatonin_mid | Daily irritable_1 | ^‡^ 0.045 | 0.559 | 0.636 |
| Melatonin_mid | BT weekday_6 | ^‡^ 0.116 | 0.156 | 0.228 |
| Melatonin_mid | BT holiday_6 | ^‡^ 0.125 | 0.176 | 0.249 |
| Melatonin_mid | BT holiday_12 | ^‡^ 0.261 | 0.006 | 0.015 |
| K6_late | SLP night_6 | ^†^ -0.155 | 0.089 | 0.143 |
| K6_late | SLP nap_6 | ^†^ -0.112 | 0.135 | 0.201 |
| K6_late | Short sleep_1 | ^†^ 0.005 | 0.956 | 0.977 |
| K6_late | Midwake_6 | ^†^ 0.341 | 0.002 | 0.006 |
| K6_late | MIBS-J_1 | ^†^ 0.284 | < 0.001 | < 0.001 |
| K6_late | M-CHAT | ^†^ 0.288 | 0.006 | 0.014 |
| K6_late | K6_1 | ^†^ 0.583 | < 0.001 | < 0.001 |
| K6_late | Fuzzy_1 | ^†^ -0.033 | 0.672 | 0.744 |
| K6_late | FA long_6 | ^†^ 0.056 | 0.511 | 0.590 |
| K6_late | FA long_1 | ^†^ 0.111 | 0.184 | 0.257 |
| K6_late | FA irritable_6 | ^†^ 0.145 | 0.049 | 0.086 |
| K6_late | Daily irritable_1 | ^†^ 0.097 | 0.207 | 0.280 |
| K6_late | BT weekday_6 | ^†^ 0.055 | 0.499 | 0.582 |
| K6_late | BT holiday_6 | ^†^ 0.019 | 0.825 | 0.874 |
| K6_late | BT holiday_12 | ^†^ 0.057 | 0.502 | 0.583 |
| K6_1 | SLP night_6 | ^†^ -0.182 | 0.006 | 0.015 |
| K6_1 | SLP nap_6 | ^†^ -0.143 | 0.062 | 0.105 |
| K6_1 | Midwake_6 | ^†^ 0.194 | 0.058 | 0.098 |
| K6_1 | MIBS-J_1 | ^†^ 0.596 | < 0.001 | < 0.001 |
| K6_1 | M-CHAT | ^†^ 0.236 | 0.020 | 0.042 |
| K6_1 | FA long_6 | ^†^ 0.161 | 0.045 | 0.081 |
| K6_1 | FA irritable_6 | ^†^ 0.088 | 0.196 | 0.270 |
| K6_1 | BT weekday_6 | ^†^ 0.148 | 0.041 | 0.075 |
| K6_1 | BT holiday_6 | ^†^ 0.123 | 0.095 | 0.150 |
| K6_1 | BT holiday_12 | ^†^ -0.021 | 0.821 | 0.874 |
| IL-6_birth | SLP night_6 | ^‡^ -0.277 | < 0.001 | < 0.001 |
| IL-6_birth | SLP nap_6 | ^‡^ 0.076 | 0.300 | 0.383 |
| IL-6_birth | Short sleep_1 | ^‡^ 0.119 | 0.030 | 0.058 |
| IL-6_birth | Midwake_6 | ^‡^ -0.209 | 0.605 | 0.681 |
| IL-6_birth | MIBS-J_1 | ^‡^ -0.018 | 0.905 | 0.941 |
| IL-6_birth | M-CHAT | ^‡^ -0.362 | < 0.001 | < 0.001 |
| IL-6_birth | K6_late | ^‡^ -0.258 | < 0.001 | < 0.001 |
| IL-6_birth | K6_1 | ^‡^ 0.103 | < 0.001 | < 0.001 |
| IL-6_birth | Fuzzy_1 | ^‡^ 0.118 | < 0.001 | < 0.001 |
| IL-6_birth | FA long_6 | ^‡^ 0.001 | 0.993 | 0.993 |
| IL-6_birth | FA long_1 | ^‡^ 0.133 | < 0.001 | < 0.001 |
| IL-6_birth | FA irritable_6 | ^‡^ -0.008 | 0.874 | 0.921 |
| IL-6_birth | EPDS_late | ^‡^ 0.042 | 0.045 | 0.081 |
| IL-6_birth | Daily irritable_1 | ^‡^ 0.170 | < 0.001 | < 0.001 |
| IL-6_birth | BT weekday_6 | ^‡^ -0.232 | < 0.001 | < 0.001 |
| IL-6_birth | BT holiday_6 | ^‡^ -0.118 | < 0.001 | < 0.001 |
| IL-6_birth | BT holiday_12 | ^‡^ -0.277 | < 0.001 | < 0.001 |
| IL-1β_mid | SLP night_6 | ^‡^ -0.254 | < 0.001 | < 0.001 |
| IL-1β_mid | SLP nap_6 | ^‡^ 0.240 | < 0.001 | < 0.001 |
| IL-1β_mid | Short sleep_1 | ^‡^ -0.361 | < 0.001 | < 0.001 |
| IL-1β_mid | Midwake_6 | ^‡^ -0.217 | 0.456 | 0.538 |
| IL-1β_mid | MIBS-J_1 | ^‡^ 0.191 | < 0.001 | < 0.001 |
| IL-1β_mid | M-CHAT | ^‡^ -0.278 | < 0.001 | < 0.001 |
| IL-1β_mid | K6_late | ^‡^ -0.114 | 0.074 | 0.122 |
| IL-1β_mid | K6_1 | ^‡^ 0.225 | < 0.001 | < 0.001 |
| IL-1β_mid | IL-6_birth | ^*^ -0.016 | 0.985 | 0.993 |
| IL-1β_mid | IL-1β_birth | ^*^ -0.014 | 0.990 | 0.993 |
| IL-1β_mid | Fuzzy_1 | ^‡^ 0.235 | < 0.001 | < 0.001 |
| IL-1β_mid | FA long_6 | ^‡^ -0.123 | < 0.001 | < 0.001 |
| IL-1β_mid | FA long_1 | ^‡^ 0.131 | < 0.001 | < 0.001 |
| IL-1β_mid | FA irritable_6 | ^‡^ 0.202 | < 0.001 | < 0.001 |
| IL-1β_mid | EPDS_late | ^‡^ -0.160 | < 0.001 | < 0.001 |
| IL-1β_mid | Daily irritable_1 | ^‡^ -0.048 | 0.001 | 0.003 |
| IL-1β_mid | BT weekday_6 | ^‡^ 0.169 | < 0.001 | < 0.001 |
| IL-1β_mid | BT holiday_6 | ^‡^ 0.192 | < 0.001 | < 0.001 |
| IL-1β_mid | BT holiday_12 | ^‡^ -0.215 | < 0.001 | < 0.001 |
| Fuzzy_1 | SLP night_6 | ^†^ -0.164 | 0.044 | 0.080 |
| Fuzzy_1 | SLP nap_6 | ^†^ -0.189 | 0.037 | 0.070 |
| Fuzzy_1 | Short sleep_1 | ^†^ 0.391 | < 0.001 | < 0.001 |
| Fuzzy_1 | Midwake_6 | ^†^ 0.264 | 0.036 | 0.069 |
| Fuzzy_1 | MIBS-J_1 | ^†^ 0.313 | < 0.001 | < 0.001 |
| Fuzzy_1 | M-CHAT | ^†^ 0.256 | 0.006 | 0.014 |
| Fuzzy_1 | K6_1 | ^†^ 0.336 | < 0.001 | < 0.001 |
| Fuzzy_1 | FA long_6 | ^†^ 0.330 | < 0.001 | < 0.001 |
| Fuzzy_1 | FA long_1 | ^†^ 0.511 | < 0.001 | < 0.001 |
| Fuzzy_1 | FA irritable_6 | ^†^ 0.109 | 0.167 | 0.243 |
| Fuzzy_1 | Daily irritable_1 | ^†^ 0.526 | < 0.001 | < 0.001 |
| Fuzzy_1 | BT weekday_6 | ^†^ 0.145 | 0.111 | 0.171 |
| Fuzzy_1 | BT holiday_6 | ^†^ 0.172 | 0.052 | 0.091 |
| Fuzzy_1 | BT holiday_12 | ^†^ 0.078 | 0.432 | 0.519 |
| FA long_6 | Midwake_6 | ^†^ 0.529 | < 0.001 | < 0.001 |
| FA long_6 | M-CHAT | ^†^ 0.410 | < 0.001 | < 0.001 |
| FA long_6 | FA irritable_6 | ^†^ 0.224 | 0.022 | 0.047 |
| FA long_6 | BT holiday_12 | ^†^ 0.185 | 0.087 | 0.140 |
| FA long_1 | SLP night_6 | ^†^ -0.155 | 0.077 | 0.126 |
| FA long_1 | SLP nap_6 | ^†^ -0.151 | 0.113 | 0.172 |
| FA long_1 | Short sleep_1 | ^†^ 0.385 | < 0.001 | < 0.001 |
| FA long_1 | Midwake_6 | ^†^ 0.186 | 0.126 | 0.190 |
| FA long_1 | MIBS-J_1 | ^†^ 0.277 | < 0.001 | 0.001 |
| FA long_1 | M-CHAT | ^†^ 0.247 | 0.005 | 0.013 |
| FA long_1 | K6_1 | ^†^ 0.426 | < 0.001 | < 0.001 |
| FA long_1 | FA long_6 | ^†^ 0.340 | < 0.001 | 0.001 |
| FA long_1 | FA irritable_6 | ^†^ 0.112 | 0.142 | 0.21 |
| FA long_1 | Daily irritable_1 | ^†^ 0.720 | < 0.001 | < 0.001 |
| FA long_1 | BT weekday_6 | ^†^ 0.163 | 0.033 | 0.063 |
| FA long_1 | BT holiday_6 | ^†^ 0.141 | 0.065 | 0.108 |
| FA long_1 | BT holiday_12 | ^†^ 0.077 | 0.394 | 0.481 |
| FA irritable_6 | Midwake_6 | ^†^ 0.109 | 0.440 | 0.524 |
| FA irritable_6 | M-CHAT | ^†^ 0.362 | < 0.001 | < 0.001 |
| FA irritable_6 | BT holiday_12 | ^†^ 0.112 | 0.232 | 0.310 |
| EPDS_late | SLP night_6 | ^†^ -0.081 | 0.320 | 0.402 |
| EPDS_late | SLP nap_6 | ^†^ -0.199 | 0.007 | 0.017 |
| EPDS_late | Short sleep_1 | ^†^ 0.184 | 0.028 | 0.055 |
| EPDS_late | Midwake_6 | ^†^ 0.401 | < 0.001 | < 0.001 |
| EPDS_late | MIBS-J_1 | ^†^ 0.244 | < 0.001 | < 0.001 |
| EPDS_late | M-CHAT | ^†^ 0.232 | 0.004 | 0.010 |
| EPDS_late | K6_late | ^†^ 0.761 | < 0.001 | < 0.001 |
| EPDS_late | K6_1 | ^†^ 0.477 | < 0.001 | < 0.001 |
| EPDS_late | Fuzzy_1 | ^†^ 0.157 | 0.023 | 0.047 |
| EPDS_late | FA long_6 | ^†^ 0.178 | 0.034 | 0.065 |
| EPDS_late | FA long_1 | ^†^ 0.095 | 0.169 | 0.243 |
| EPDS_late | FA irritable_6 | ^†^ 0.087 | 0.268 | 0.350 |
| EPDS_late | Daily irritable_1 | ^†^ 0.101 | 0.172 | 0.246 |
| EPDS_late | BT weekday_6 | ^†^ 0.019 | 0.783 | 0.845 |
| EPDS_late | BT holiday_6 | ^†^ -0.001 | 0.990 | 0.993 |
| EPDS_late | BT holiday_12 | ^†^ 0.055 | 0.359 | 0.448 |
| Daily irritable_1 | SLP night_6 | ^†^ -0.250 | 0.007 | 0.016 |
| Daily irritable_1 | SLP nap_6 | ^†^ -0.204 | 0.024 | 0.049 |
| Daily irritable_1 | Short sleep_1 | ^†^ 0.540 | < 0.001 | < 0.001 |
| Daily irritable_1 | Midwake_6 | ^†^ 0.186 | 0.055 | 0.095 |
| Daily irritable_1 | MIBS-J_1 | ^†^ 0.341 | < 0.001 | < 0.001 |
| Daily irritable_1 | M-CHAT | ^†^ 0.203 | 0.010 | 0.022 |
| Daily irritable_1 | K6_1 | ^†^ 0.422 | < 0.001 | < 0.001 |
| Daily irritable_1 | FA long_6 | ^†^ 0.267 | 0.004 | 0.010 |
| Daily irritable_1 | FA irritable_6 | ^†^ 0.127 | 0.101 | 0.158 |
| Daily irritable_1 | BT weekday_6 | ^†^ 0.224 | 0.010 | 0.022 |
| Daily irritable_1 | BT holiday_6 | ^†^ 0.205 | 0.021 | 0.044 |
| Daily irritable_1 | BT holiday_12 | ^†^ 0.099 | 0.240 | 0.318 |
| BT weekday_6 | Midwake_6 | ^†^ -0.142 | 0.300 | 0.383 |
| BT weekday_6 | M-CHAT | ^†^ 0.309 | < 0.001 | < 0.001 |
| BT weekday_6 | FA long_6 | ^†^ 0.186 | 0.070 | 0.116 |
| BT weekday_6 | FA irritable_6 | ^†^ 0.117 | 0.105 | 0.163 |
| BT weekday_6 | BT holiday_6 | ^†^ 0.995 | < 0.001 | < 0.001 |
| BT weekday_6 | BT holiday_12 | ^†^ 0.695 | < 0.001 | < 0.001 |
| BT holiday_6 | Midwake_6 | ^†^ -0.117 | 0.384 | 0.473 |
| BT holiday_6 | M-CHAT | ^†^ 0.330 | < 0.001 | < 0.001 |
| BT holiday_6 | FA long_6 | ^†^ 0.143 | 0.169 | 0.243 |
| BT holiday_6 | FA irritable_6 | ^†^ 0.162 | 0.029 | 0.057 |
| BT holiday_6 | BT holiday_12 | ^†^ 0.711 | < 0.001 | < 0.001 |
| BT holiday_12 | M-CHAT | ^†^ 0.448 | < 0.001 | < 0.001 |

^†^ Polychoric correlation was used for ordinal variable pairs.

^‡^ Polyserial correlation was used for ordinal and continuous variable pairs.

^*^ Pearson correlation was used for continuous variable pairs.
