# Supplementary material for: Multilevel Factors and Indicators of Atypical Neurodevelopment During Early Infancy in Japan: Prospective, Longitudinal, Observational Study
Source: JMIR Pediatr Parent. 2025 Apr 4;8:e58337. doi: 10.2196/58337 (PMC11990654; doi:10.2196/58337)
Supplement: Multimedia Appendix 6 [file pediatrics-v8-e58337-s006.docx]

To evaluate the longitudinal factors associated with Developmental Diversity (DD) in infants, we conducted feature importance analysis using SHAP values on a logistic regression model with L2 regularization to predict DD-positive cases. The optimal threshold was determined using the Youden index. Figure S1 illustrates the ROC and PR curves, while Figure S2 presents the confusion matrix at the optimal threshold for this model. TableS1 shows a comparison of the performance of different hyperparameter combinations in the logistic regression model. Table S2 presents the standard statistics and the results of the inferential tests of the model.

### Figure S1. (A) Receiver operating characteristic (ROC) curve and (B) Precision-Recall (PR) curve of the logistic regression model in the test set.


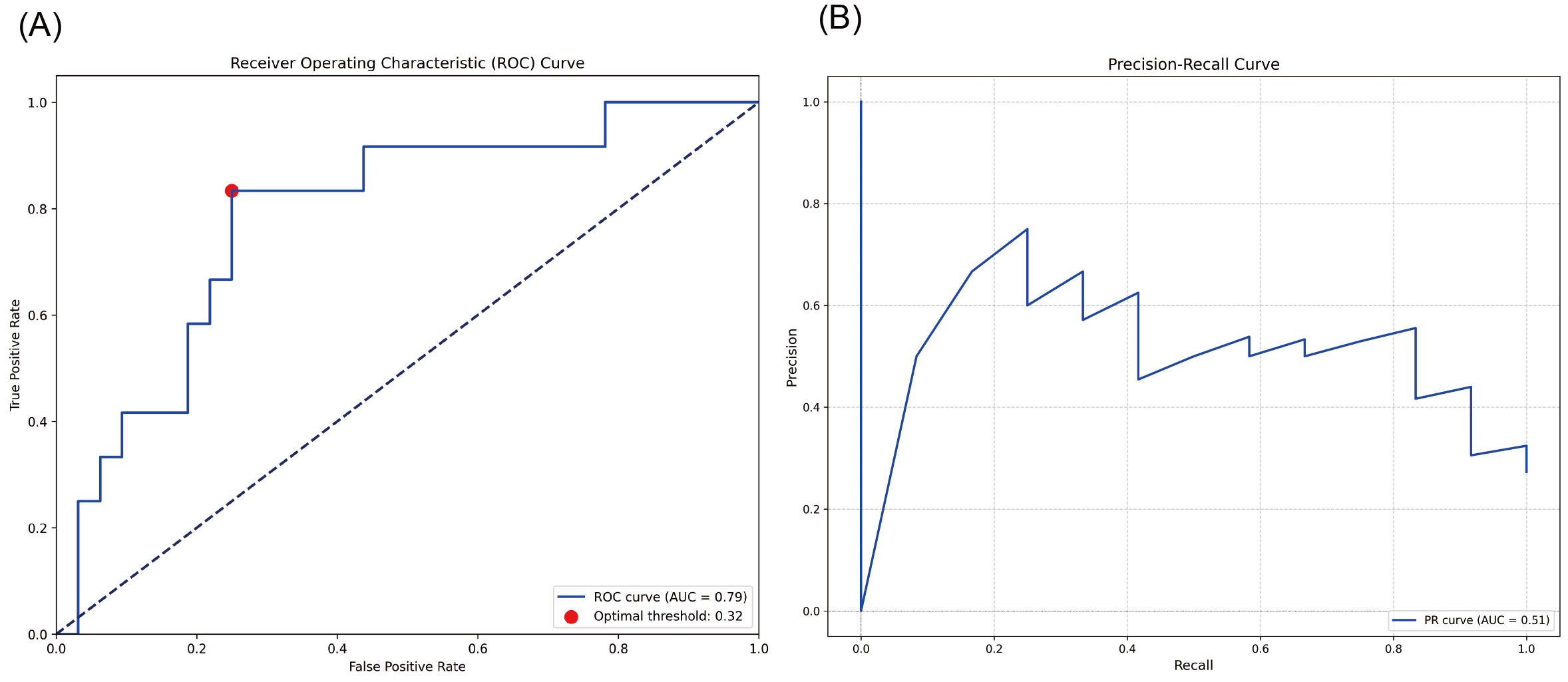


### Figure S2. Confusion matrix of the logistic regression model at the optimal threshold.


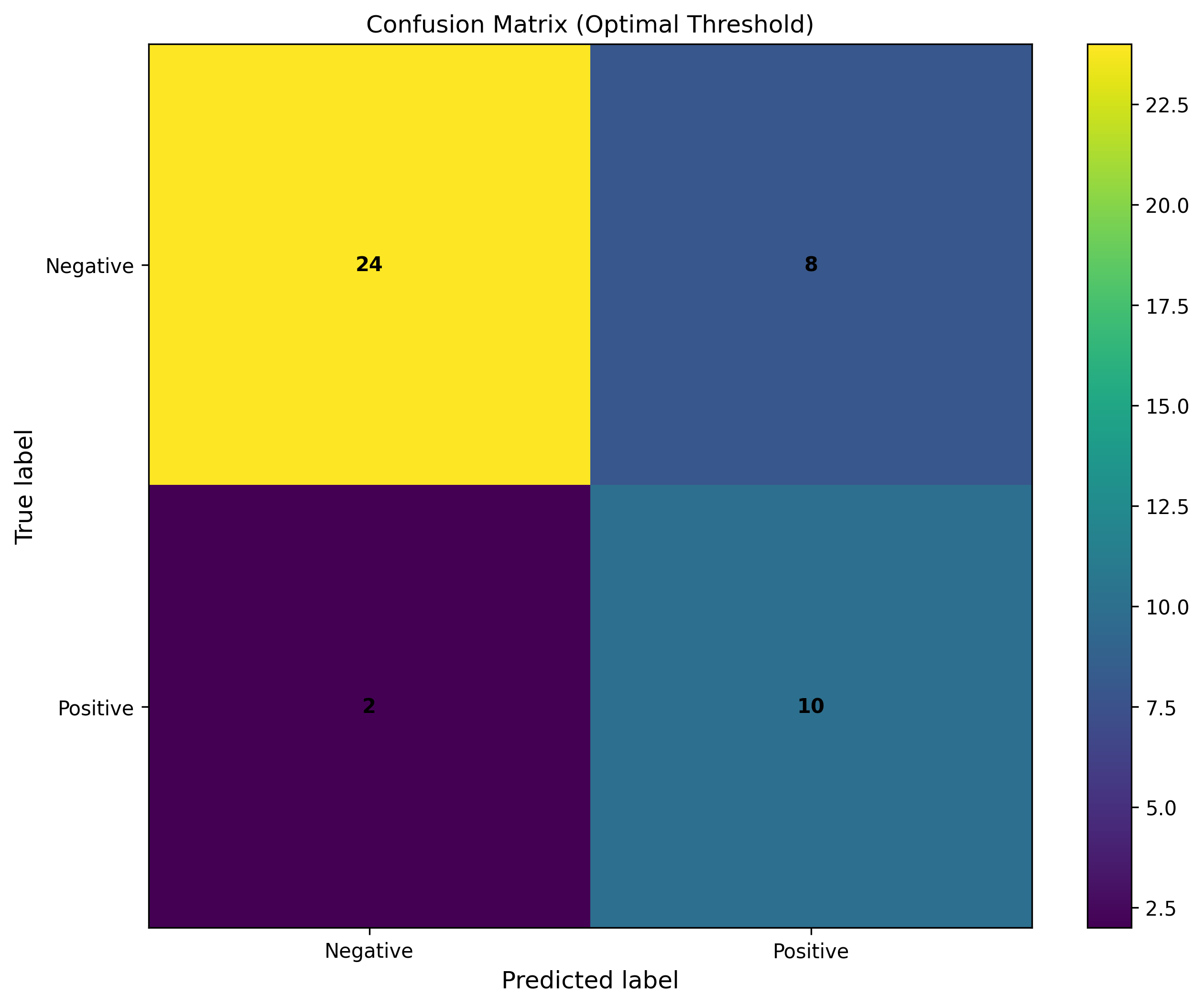


### Table S1. Performance comparison of various hyperparameter combinations in logistic regression models.

|  | penalty | None | | | | | l1 | | | | | l2 | | | | |
| --- | --- | --- | --- | --- | --- | --- | --- | --- | --- | --- | --- | --- | --- | --- | --- | --- |
|  | solver | newton-cg | lbfgs | liblinear | sag | saga | newton-cg | lbfgs | liblinear | sag | saga | newton-cg | lbfgs | liblinear | sag | saga |
| C | 1.00E-05 | 0.64 | 0.64 |  | 0.64 | 0.64 |  |  | 0.73 |  | 0.73 | 0.73 | 0.73 | 0.67 | 0.73 | 0.73 |
|  | 0.0001 | 0.64 | 0.64 |  | 0.64 | 0.64 |  |  | 0.73 |  | 0.73 | 0.73 | 0.73 | 0.67 | 0.73 | 0.73 |
|  | 0.001 | 0.64 | 0.64 |  | 0.64 | 0.64 |  |  | 0.73 |  | 0.73 | 0.73 | 0.73 | 0.66 | 0.73 | 0.73 |
|  | 0.01 | 0.64 | 0.64 |  | 0.64 | 0.64 |  |  | 0.73 |  | 0.73 | 0.73 | 0.73 | 0.67 | 0.73 | 0.73 |
|  | 0.1 | 0.64 | 0.64 |  | 0.64 | 0.64 |  |  | 0.73 |  | 0.73 | 0.69 | 0.69 | 0.73 | 0.69 | 0.69 |
|  | 1 | 0.64 | 0.64 |  | 0.64 | 0.64 |  |  | 0.7 |  | 0.69 | 0.69 | 0.69 | 0.7 | 0.69 | 0.69 |
|  | 10 | 0.64 | 0.64 |  | 0.64 | 0.64 |  |  | 0.65 |  | 0.65 | 0.64 | 0.64 | 0.64 | 0.64 | 0.64 |
|  | 100 | 0.64 | 0.64 |  | 0.64 | 0.64 |  |  | 0.64 |  | 0.64 | 0.64 | 0.64 | 0.64 | 0.64 | 0.64 |
|  | 1000 | 0.64 | 0.64 |  | 0.64 | 0.64 |  |  | 0.64 |  | 0.64 | 0.64 | 0.64 | 0.64 | 0.64 | 0.64 |
|  | 10000 | 0.64 | 0.64 |  | 0.64 | 0.64 |  |  | 0.64 |  | 0.64 | 0.64 | 0.64 | 0.64 | 0.64 | 0.64 |
|  | 100000 | 0.64 | 0.64 |  | 0.64 | 0.64 |  |  | 0.64 |  | 0.64 | 0.64 | 0.64 | 0.64 | 0.64 | 0.64 |

### Table S1. (Continued.)

|  |  | elasticnet | | | | | | | | | | |
| --- | --- | --- | --- | --- | --- | --- | --- | --- | --- | --- | --- | --- |
|  | l1_ratio | 0 | 0.1 | 0.2 | 0.3 | 0.4 | 0.5 | 0.6 | 0.7 | 0.8 | 0.9 | 1 |
| C | 1.00E-05 | 0.73 | 0.73 | 0.73 | 0.73 | 0.73 | 0.73 | 0.73 | 0.73 | 0.73 | 0.73 | 0.73 |
|  | 0.0001 | 0.73 | 0.73 | 0.73 | 0.73 | 0.73 | 0.73 | 0.73 | 0.73 | 0.73 | 0.73 | 0.73 |
|  | 0.001 | 0.73 | 0.73 | 0.73 | 0.73 | 0.73 | 0.73 | 0.73 | 0.73 | 0.73 | 0.73 | 0.73 |
|  | 0.01 | 0.73 | 0.73 | 0.73 | 0.73 | 0.73 | 0.73 | 0.73 | 0.73 | 0.73 | 0.73 | 0.73 |
|  | 0.1 | 0.69 | 0.7 | 0.7 | 0.73 | 0.73 | 0.73 | 0.73 | 0.73 | 0.73 | 0.73 | 0.73 |
|  | 1 | 0.69 | 0.69 | 0.69 | 0.69 | 0.69 | 0.69 | 0.69 | 0.68 | 0.69 | 0.69 | 0.69 |
|  | 10 | 0.64 | 0.64 | 0.64 | 0.64 | 0.64 | 0.65 | 0.65 | 0.65 | 0.65 | 0.65 | 0.65 |
|  | 100 | 0.64 | 0.64 | 0.64 | 0.64 | 0.64 | 0.64 | 0.64 | 0.64 | 0.64 | 0.64 | 0.64 |
|  | 1000 | 0.64 | 0.64 | 0.64 | 0.64 | 0.64 | 0.64 | 0.64 | 0.64 | 0.64 | 0.64 | 0.64 |
|  | 10000 | 0.64 | 0.64 | 0.64 | 0.64 | 0.64 | 0.64 | 0.64 | 0.64 | 0.64 | 0.64 | 0.64 |
|  | 100000 | 0.64 | 0.64 | 0.64 | 0.64 | 0.64 | 0.64 | 0.64 | 0.64 | 0.64 | 0.64 | 0.64 |

### Table S2. Standard statistics and inferential test results for the logistic regression model.

| Feature | Coefficient | Std Error | z-value | p-value |
| --- | --- | --- | --- | --- |
| Mel_mid | 1.058745495 | 0.778476132 | 1.360023064 | 0.173822625 |
| IL1b_mid | 0.322875695 | 2.021993716 | 0.159681849 | 0.8731317 |
| IL1b_uv | 0.019219651 | 0.468502214 | 0.041023609 | 0.967277075 |
| IL6_uv | 0.039907339 | 0.325533124 | 0.122590718 | 0.902431205 |
| EPDS_late | 0.111477362 | 0.048481257 | 2.29939089 | 0.021482753 |
| K6_late | 0.125535526 | 0.054407338 | 2.307327123 | 0.021036591 |
| troublesome_1 | 0.266569376 | 0.186625569 | 1.428364705 | 0.153186913 |
| badFA_1 | 0.245754257 | 0.177691511 | 1.383038812 | 0.166652965 |
| crying_1 | 0.23995413 | 0.200856775 | 1.194652904 | 0.23222267 |
| short_1 | 0.367200347 | 0.193870931 | 1.894045408 | 0.058218983 |
| K6_1 | 0.124747938 | 0.053320859 | 2.339571038 | 0.0193059 |
| MIBS_J | 0.175689822 | 0.076216098 | 2.305153712 | 0.021157966 |
| SLP_night_6 | -0.531725899 | 0.223010967 | -2.384303812 | 0.017111469 |
| SLP_nap_6 | -0.654537389 | 0.352579769 | -1.856423558 | 0.063393209 |
| bedtime_WD_6 | 0.564087685 | 0.209796421 | 2.688738363 | 0.007172261 |
| bedtime_HD_6 | 0.54861748 | 0.206771277 | 2.653257683 | 0.007971899 |
| FA_long_6 | 0.79236423 | 0.262874846 | 3.014226135 | 0.002576357 |
| FA_grumpy_sli_6 | 0.519473536 | 0.162748011 | 3.191888701 | 0.001413458 |
| resleeping_6 | 0.540409254 | 0.403696393 | 1.338652671 | 0.180683771 |
| bedtime_HD_12 | 0.682758012 | 0.210483537 | 3.243759688 | 0.001179632 |
